# Sensitivity of Riparian Buffer Designs to Climate Change—Nutrient and Sediment Loading to Streams: A Case Study in the Albemarle-Pamlico River Basins (USA) Using HAWQS

**DOI:** 10.3390/su132212380

**Published:** 2021-11-09

**Authors:** Santosh R. Ghimire, Joel Corona, Rajbir Parmar, Gouri Mahadwar, Raghavan Srinivasan, Katie Mendoza, John M. Johnston

**Affiliations:** 1 U.S. Environmental Protection Agency Office of Research and Development, Athens, GA 30605, USA; 2 U.S. Environmental Protection Agency Office of Water, Washington, DC 20460, USA; 3 Former U.S. Environmental Protection Agency ORISE Participant, Oak Ridge, TN 37831-0117, USA; 4 Blackland Research and Extension Center and Department of Ecology and Conservation Biology, Texas A&M Agrilife Research and Texas A&M University, Temple, TX 76502, USA

**Keywords:** riparian buffer zone, watershed, water quality, sustainability, ecosystem services

## Abstract

Riparian buffer zones (RBZs) provide multiple benefits to watershed ecosystems. We aimed to conduct an extensive sensitivity analysis of the RBZ designs to climate change nutrient and sediment loadings to streams. We designed 135 simulation scenarios starting with the six baselines RBZs (grass, urban, two-zone forest, three-zone forest, wildlife, and naturalized) in three 12-digit Hydrologic Unit Code watersheds within the Albemarle-Pamlico river basin (USA). Using the hydrologic and water quality system (HAWQS), we assessed the sensitivity of the designs to five water quality indicator (WQI) parameters: dissolved oxygen (DO), total phosphorous (TP), total nitrogen (TN), sediment (SD), and biochemical oxygen demand (BD). To understand the climate mitigation potential of RBZs, we identified a subset of future climate change projection models of air temperature and precipitation using EPA’s Locating and Selecting Scenarios Online tool. Analyses revealed optimal RBZ designs for the three watersheds. In terms of watershed ecosystem services sustainability, the optimal Urban RBZ in contemporary climate (1983–2018) reduced SD from 61–96%, TN from 34–55%, TP from 9–48%, and BD from 53–99%, and raised DO from 4–10% with respect to No-RBZ in the three watersheds. The late century’s (2070–2099) extreme mean annual climate changes significantly increased the projected SD and BD; however, the addition of urban RBZs was projected to offset the climate change reducing SD from 28–94% and BD from 69–93% in the watersheds. All other types of RBZs are also projected to fully mitigate the climate change impacts on WQI parameters except three-zone RBZ.

## 1. Introduction

According to the 2017 National Water Quality Inventory Report to the U.S. Congress, 46% of river and stream miles, 21% of lakes, ponds, and reservoirs, 18% of the coastal and Great Lakes waters, and 32% of wetland areas are in poor biological condition [1]. Nonpoint sources are responsible for at least half of all water pollution contributors, such as sediments, nutrients, pathogenic bacteria, pesticides, acid rain, and polychlorinated biphenyls (PCBs); nationwide, agricultural lands contribute nearly 70% of the total loads of nitrogen and phosphorus each year [2]. Unsustainable activities, such as rapid urbanization and large-scale agricultural practices, increase sediments, nutrients (e.g., nitrogen and phosphorus forms), and chemicals (e.g., herbicides and pesticides) and deplete dissolved oxygen, impacting stream water quality and biodiversity [3–9]. Projected climate changes will directly or indirectly exacerbate these impacts due to the frequency and duration of droughts, increased global temperature, and heavy storm events [10].

Riparian buffer zones (RBZs) represent best management practices (BMPs) that can provide multiple benefits to watershed ecosystems. RBZs perform this by controlling the number of sediments and nutrients entering from non-point sources, filtering polluted air from agrochemically treated local farms, providing habitat for diverse organisms, and enhancing woodland connectivity corridors [8,9,11,12]. Consequently, the U.S. government provides various riparian restoration and preservation programs to landowners under the 1996 Farm Bill, including the continuous Conservation Reserve Program (CRP), Environmental Quality Incentives Program (EQIP), Wildlife Habitat Incentives Program (WHIP), Wetlands Reserve Program (WRP), Stewardship Incentives Program (SIP), Emergency Watershed Protection Program (EWP), and the National Resources Conservation Service (NRCS) National Conservation Buffers Initiative [13].

Globally, several studies addressed the riparian buffers’ impacts on sediment and nutrient removal effectiveness [6,7,11,14,15]. Others studied riparian ecosystems, nutrient biogeochemical and hydrological processing, riparian buffer function, and modeling techniques [16–19]. However, effectiveness varies with buffer design (i.e., varying buffer vegetation and width), site-specific factors, such as buffer management, agricultural practices (crop rotations and fertilization), pollutant properties, biological processes, and the condition of riparian areas (hydrology, vegetation, and geomorphology) [9,12,15,20,21]. While some literature indicated that narrow, continuous buffers were more effective than intermittent, wide buffers, others found that narrow buffers contributed nitrogen to riparian zones [7,9,12,14]. A meta-analysis of 46 global studies published between 1980 and 2017 indicated a variation in nitrogen removal from 20–100% [14]. Another meta-analysis of 73 studies reported that a 20 m buffer removed approximately 91–100% of nitrogen and 97–100% of phosphorous [22]. Others reported that wetland buffer zone effectiveness varied from 12–80% of surface water nitrogen removal to 95% nitrate reductions in the ground water [12]. A study conducted in Finland, Norway, Sweden, and Denmark showed that buffer zones and wetlands decreased the total phosphorous loads from agricultural runoff from 27–97% [23]. In North Carolina (USA), riparian buffers’ ground water nitrate removal efficiency ranged from 67–100% [24] and sediment trapping ranged from 60–90% [25,26]. In Nebraska (USA), the buffer strips reduced total phosphorous from 55–79% [27]. In Virginia (USA), orchard grass filter strips trapped 84% of the sediment and soluble solids from surface runoff [28]. In Maryland, North Carolina, and Georgia (USA), the application of riparian forest buffers in coastal plains reduced 67–89% of nitrogen inputs [29]. A study conducted in the Chesapeake Bay (USA) reported that, on average, buffers in Coastal Plain watersheds had a higher relative nitrate removal potential (95%) than Piedmont buffers (35%) and Appalachian Mountain watersheds (39%) [30]. Riparian buffer efficiency has been studied for over 30 years, yet the areas of greatest debate are still buffered width and vegetation (grassy versus woody) [7,12,14,31,32] (Appendix A, Table A1).

### Objectives, Scope, and Novel Contribution

This study focuses on protecting stream water quality and restoring impaired waters under the 303(d) section of the Clean Water Act (CWA) [33]; the CWA aims to “restore and maintain the chemical, physical, and biological integrity of the Nation’s waters” (CWA, Section 101 (a) [34]). Our objective was to conduct extensive sensitivity analysis of the riparian buffer designs (i.e., varying buffer vegetation and width) adapted to three watersheds within the Albemarle-Pamlico river basin (USA) to understand the potential tradeoffs between the designs and stream water quality. We also integrated future climate projections (temperature and precipitation) into the analysis. We compared the potential tradeoffs in terms of five water quality indicator (WQI) parameters: dissolved oxygen (DO) (mg/L), total phosphorous (TP) (mg/L), total nitrogen (TN) (mg/L), total suspended solids as sediment (SD) (mg/L), and biochemical oxygen demand (BD) (mg/L). The terms riparian buffer, riparian zone, buffer zone, buffer strip, filter strip, and vegetated filter strip are defined differently depending on the application [12]; however, we used the term “riparian buffer zone” (RBZ) to represent the zone of vegetation adjacent to streams, rivers, creeks, lakes, wetlands, or other interconnected inland aquatic systems.

We advanced the riparian buffer science tailored to local conditions within the Albemarle-Pamlico river basins by demonstrating sensitivity simulations of RBZs for representative southeastern U.S. watersheds—which, to our knowledge, has not been done. Our study provides a firm basis for future RBZ modeling in other watersheds, which is necessary for appropriate RBZ decision making. The following sections describe methods, tools, and results, with concluding remarks on study implications.

## 2. Material and Methods

**Figure 1.**
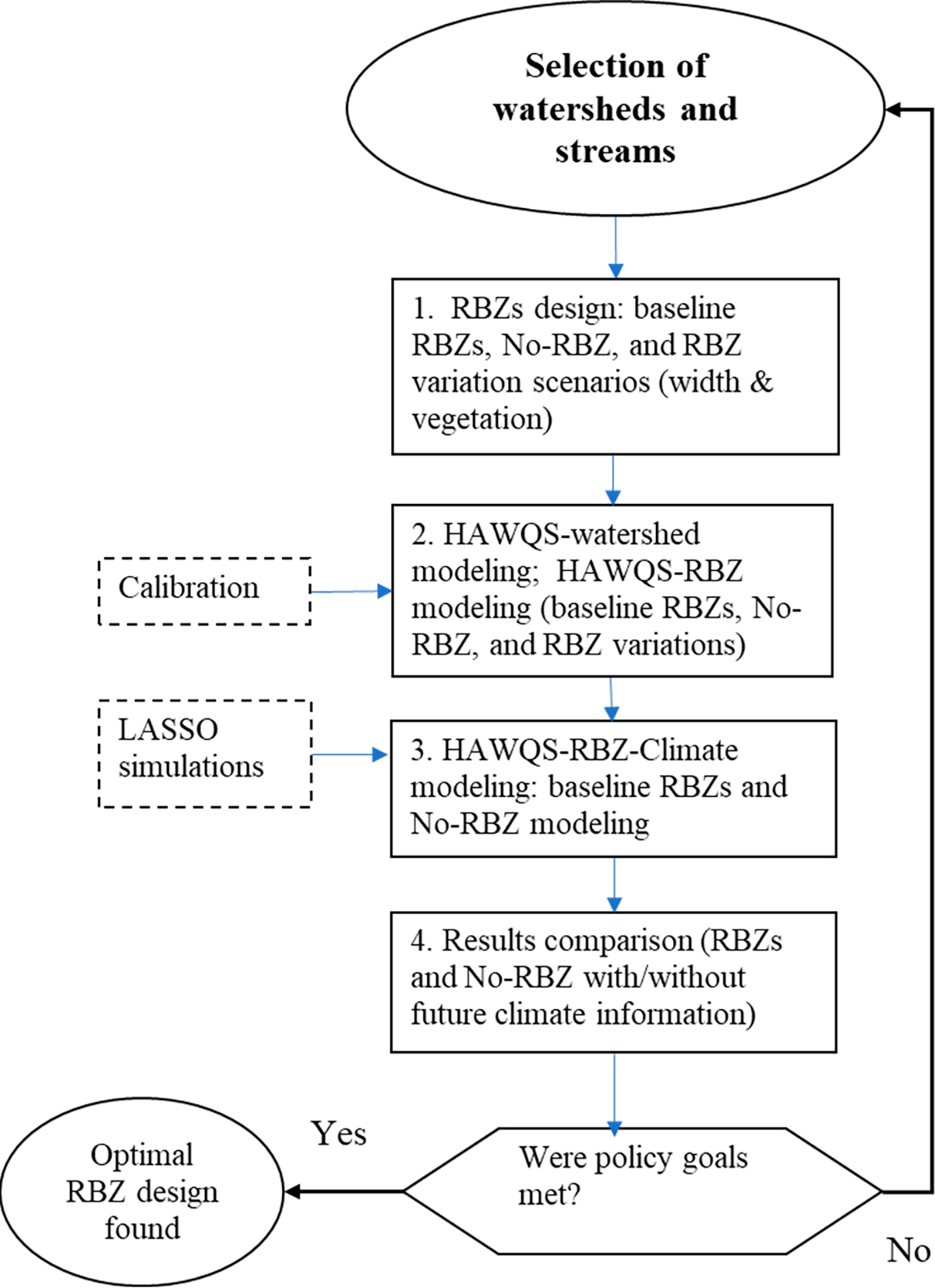
Flow diagram depicting the riparian buffer zone (RBZ) sensitivity analysis. The number of RBZ designs varies with the watershed land use types.

The material and methods are described below and depicted in Figure 1.

### 2.1. Watershed Selection

**Figure 2.**
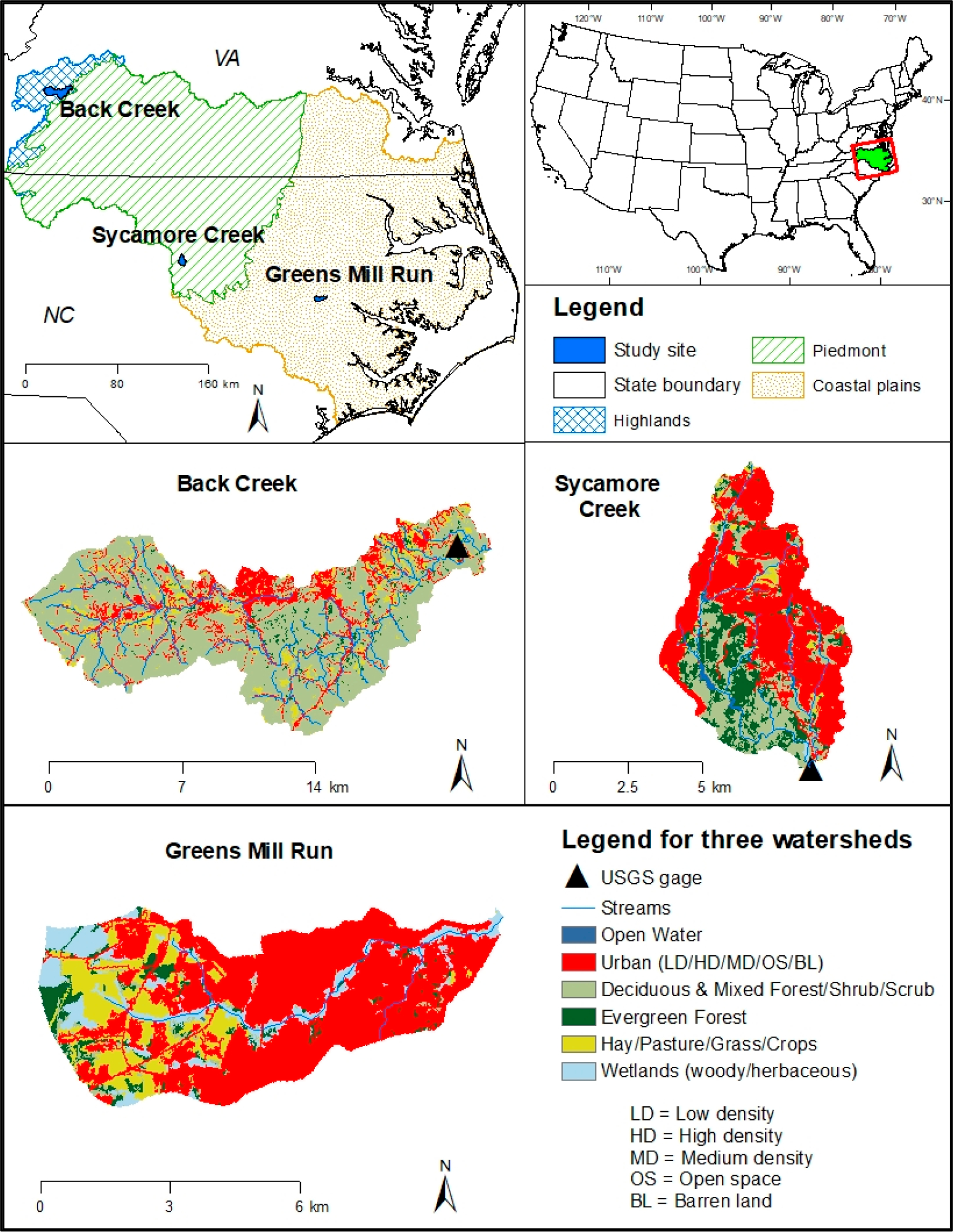
Studied watersheds, land use types, and USGS stream gage locations within Albemarle-Pamlico River Basin (after Ghimire and Johnston [3]). Land use types were based on the National Land Cover Database (NLCD) 2016 [39], which cross-references the National Wetland Inventory (NWI) and the National Agricultural Statistics Service (NASS) [40] Crop Data Layer (CDL) with a 30-m pixel resolution.

We selected three watersheds (12-digit Hydrologic Unit Codes, HUCs) from previously studied Albemarle-Pamlico river basins situated in North Carolina (NC) and Virginia (VA) USA (Figure 2). The three watersheds were Back Creek (HUC # 030101010405; VA), Sycamore Creek (HUC # 030202010802; NC), and Greens Mill Run (HUC # 030201030403; NC) [35–37]. The entire 52,000-km^2^ Albemarle-Pamlico is the second largest estuarine complex in the lower 48 United States [38]. The selected three watersheds have varying land use types, elevation, and population densities from highlands, VA to coastal plains, NC. The largest, Back Creek (152 km^2^), has the smallest urban area (19%); while the smallest, Greens Mill Run (34 km^2^), has the greatest urban area (70%).

### 2.2. RBZs Design

We established six types of baselines (i.e., starting points) RBZ designs (Table 1) and conceptualized 135 RBZ simulation scenarios as described in Table 2. We also constructed the No-RBZ scenario of zero buffer width (0 m). The baseline designs were created based on available data for the various types of riparian buffer designs (width and vegetation) published in the literature [41] (Appendix A Table A1). For each baseline design, we created an additional 8 scenarios (baseline buffer width ±100% at ±25% variation) for the sensitivity modeling, including the baseline (average width) and No-RBZs (zero width) (Table 2). While the width variation (Table 2) was conceptualized based on the literature data, the RBZ vegetation represented the actual study sites’ (watersheds) land use types (Table 3). The total number of scenarios for each watershed varied with land use types.

### 2.3. HAWQS Watershed Modeling and Calibration

We applied the Hydrologic and Water Quality System (HAWQS, version 1.2) [42], a web-based modeling platform that employed a core modeling engine called the Soil and Water Assessment Tool (SWAT) [43], to assess water quality impacts at the watershed outlets. Using HAWQS, we initially developed models for each watershed with weather dataset from 1981–2018. The initial HAWQS watershed models involved the Hargreaves evapotranspiration method and the parameter-elevation regressions on independent slopes model (PRISM) [44], daily weather input data for 36 years (from 1981–2018) with a 2-year spin-up period (hereafter referred to as contemporary climate).

SWAT, developed and maintained by the U.S. Department of Agriculture and Texas A&M University since the 1990s, is a widely used comprehensive (requiring diversity of information) watershed modeling tool for simulating the quality and quantity of surface and ground water [35,45]. The most recent version, SWAT2012 rev. 681 (2020) [46], provides two methods for representing riparian buffers in the form of width of the edge of the filter strip (FILTERW) and the vegetation filter strip (VFS) in the management operations file (.ops); for details, see the ArcSWAT users’ guide [47], SWAT theoretical documentation [48], and conservation practice modeling guide for SWAT [49]. The current version of HAWQS (version 1.2) utilizes the FILTERW method to represent riparian buffers.

HAWQS (version 1.2) uses the National Hydrology Dataset Plus (NHDPlus) from 2010, Crop Data Layer (CDL) from 2011–2012, and National Land Cover Dataset (NLCD) from 2006 to delineate watersheds at the HUC8, HUC10, and HUC12 levels, with soil data retrieved from the State Soil Geographic (STATSGO) dataset [50]. HAWQS is configured for the contiguous U.S.; however, users need to manually calibrate non-calibrated watershed models (see the HAWQS User Guide [51] for details). As a first step in quality control of the data outputs in the process of calibration, we used the SWAT check tool to identify potential model problems. We then calibrated the initial HAWQS watershed models with the observed U.S. Geological Survey (USGS) streamflow within or nearby each watershed using SWAT Calibration and Uncertainty Programs (SWAT-CUP), a publicly available calibration tool [52]. The Nash and Sutcliffe efficiency (NSE), Kling–Gupta efficiency (KGE), and percent bias (PBIAS) were considered as model performance statistics. Model simulations were considered satisfactory for streamflow if NSE and KGE values were greater than 0.50 and the absolute magnitude of the PBIAS value was less than 25% [53]. For additional information on calibration see Appendix A (Table A3).

For Back Creek, both monthly and daily gage data (1981–2018) was obtained from the USGS (Figure 2). Sycamore Creek did not have a USGS gage location within the watershed; however, monthly data (1988–2018) from a USGS gauge station (USGS 0208726005) located in an adjacent watershed (Crabtree Creek HUC # 030202010803; Figure 2) was used. Although a gage station (USGS 02084070) was found within the Greens Mill Run, it did not have sufficient data for the calibration. The Sycamore Creek parameters were used for Greens Mill Run calibration because the land use distribution and amount of annual precipitation were similar (Table A2).

### 2.4. HAWQS–RBZ Modeling

Building upon the calibrated HAWQS watershed models with contemporary weather conditions, we set up the HAWQS–RBZ models for RBZ design scenarios and No-RBZ scenarios. We used a HAWQS functionality called FILTERW, the width of edge-of-field filter strip, to represent RBZs by varying the widths as shown in Table 2 and performed the RBZ and No-RBZ simulations. We recorded and analyzed the 36-year (1983–2018) daily (36-y average daily) simulation results of the WQI parameter concentrations for each watershed. The 2-year spin-up period (1981–1982) was not included in the analysis.

These concentrations were normalized with respect to maximum WQI parameter concentration. To quantify tradeoffs of RBZ and No-RBZ, we estimated the percentage (%) change (reduction or improvement) in WQI parameter concentration using the normalized WQI parameter value (*C*_*r*_/*C*_*n*_, the concentration ratio in Equation (1) as follows:

(1) $$Ε=\left(1-\frac{C_{r}}{C_{n}}\right)\times 100\%$$

where:

*E* = Percentage change (reduction or improvement) in WQI parameter concentration (%);

*C*_*n*_ = Maximum WQI parameter concentration due to No-RBZ (mg/L);

*C*_*r*_ = WQI parameter concentration due to RBZ application (mg/L).

### 2.5. LASSO—Climate Modeling

To understand the climate mitigation potential of RBZs, we utilized EPA’s Locating and Selecting Scenarios Online (LASSO) tool [54], and systematically assessed a subset of future climate change projection models of air temperature and precipitation for three timeframes: 2021–2050, 2041–2070, and 2070–2099 [55]. The LASSO tool bounds a range of a larger group of climate projections in two dimensions of air temperature and precipitation, simultaneously allowing an efficient selection of representative future climate change models. The LASSO simulation steps are highlighted below (also see Figure A3; Appendix A):

- Definition of a study area: U.S. EPA Region and U.S. state level data were available (excluding Alaska, Hawaii, or the American Territories).
- Selection of a data source: bias corrected spatially downscaled (BCSD) and localized constructed analogs (LOCA) datasets were available. Each of these datasets represented downscaled information (i.e., translated into higher-resolution information that can be used as input to local or regional impact analyses, from the Coupled Model Intercomparison Project 5 (CMIP5) General Circulation Models (GCMs)) [54,56,57]. The LOCA dataset requires finer spatial resolution at 1/16° than that of BCSD at 1/8°; however, no one data source is better or more accurate than the other [54].
- Selection of an emission pathway: The moderate and rising emission scenarios known as the representative concentration pathways (RCP), RCP4.5 and RCP8.5, were available. RCP8.5 refers to the rising radiative forcing pathway (i.e., cumulative measure of human emissions of greenhouse gases (GHGs) from all sources expressed in Watts per square meter (W/m^2^)), leading to 8.5 W/m^2^ in 2100, and the RCP 4.5 refers to moderate stabilization without overshoot pathway to 4.5 W/m^2^ at stabilization after 2100 [56,58]. These two are the most frequently appearing RCPs in the literature.
- Selection of climate variables: climate variables (air temperature and precipitation) for five seasons (annual, winter, spring, summer, and fall) and three timeframes (2021–2050, 2041–2070, and 2070–2099) were available.
- Climate model selection strategies: Four climate model selection strategies were available within the LASSO tool that included LASSO, four corners, middle corners, and double median. Each strategy offers a subset of future climate projection models.
- Climate projection simulation and analysis: Given the numbers of data sources, RCPs, seasons, timeframes, and selection strategies, we used the “scenario discovery” [59] approach to obtain a manageable number of representative projections of precipitation (i.e., wettest and driest precipitation) and air temperature (i.e., hottest and coldest temperature) that served as input into the HAWQS modeling analysis. In this approach, we started a LASSO climate simulation for the State of NC by choosing a combination of LOCA, RCP8.5, the LASSO strategy for all the three timeframes, and five seasons (Table A4). The LASSO strategy was selected at first because it corresponded to the lowest risk tolerance, meaning it included the largest amount of information as compared to other strategies and was recommended by the LASSO tool [54]. We downloaded the spatial data, maps, and scatterplot graphics, compared future climate projection results to each other, and determined the extreme mean values of precipitation (i.e., wettest and driest precipitation) and air temperature (i.e., hottest and coldest temperature) for NC and VA.

### 2.6. HAWQS–RBZ Climate Modeling

We created HAWQS–RBZ climate models by incorporating the extreme climate values of air temperature and precipitation identified from the subset of climate models into the calibrated baseline HAWQS–RBZ and No-RBZ models. For each watershed, we compared the projected future climate-RBZ impacts to the contemporary climate No-RBZ impacts and future climate No-RBZ impacts.

## 3. Results

### 3.1. RBZ Designs and WQI Parameters

For each of the RBZ design scenarios (Tables 1 and 2), we analyzed the sensitivity of outlet-WQI parameters (DO, TP, TN, SD, and BD) that reflected the water quality exiting the entire watershed.

### 3.2. HAWQS Watershed Model Calibration

**Figure A1.**
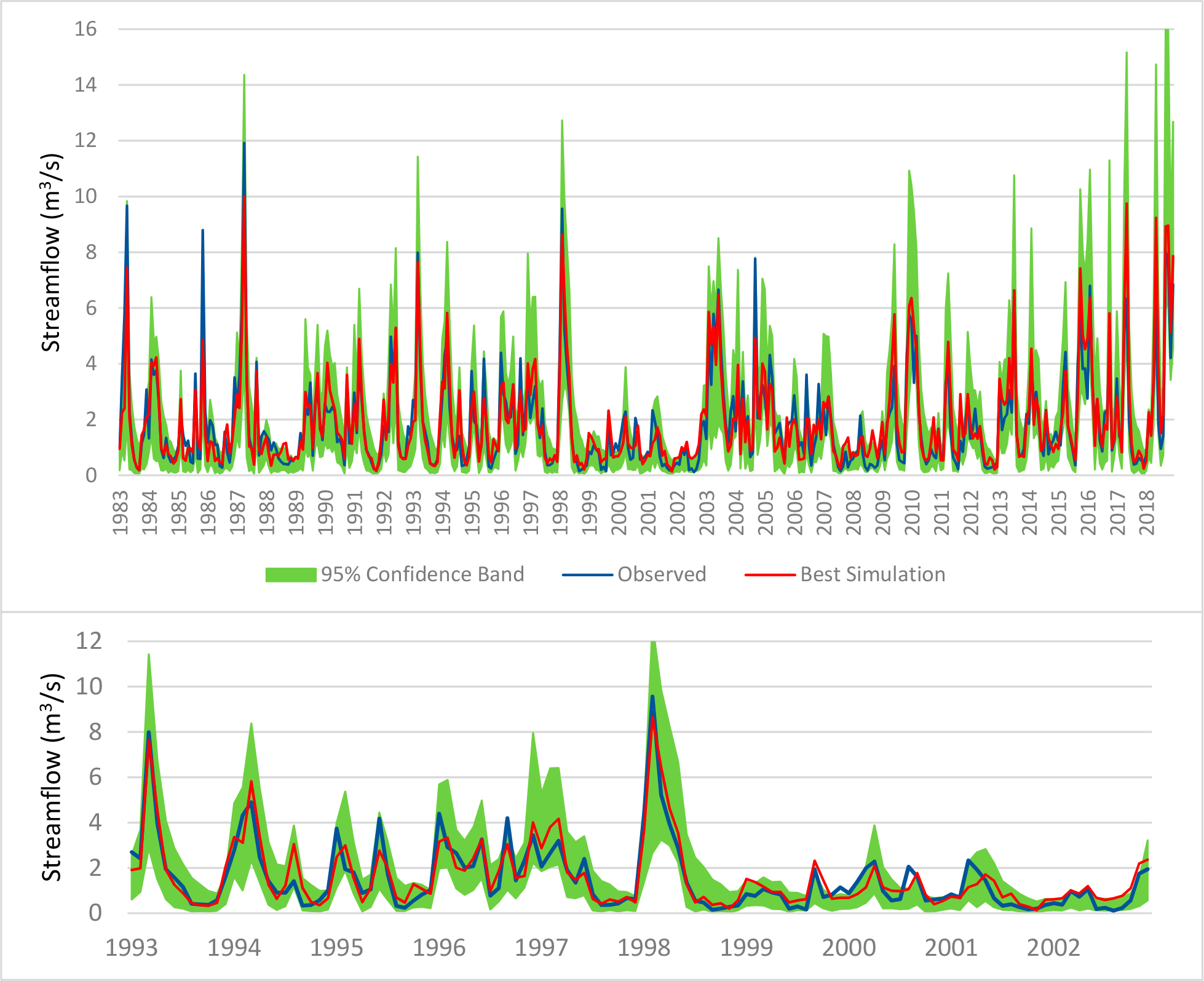
(**Top**) Back Creek watershed monthly USGS and SWAT simulated (best estimation) streamflow along with the 95% confidence range for all simulations from 1983–2018. (**Bottom**) Zoomed-in to show detail for a 10-year period from 1993–2002.

**Figure A2.**
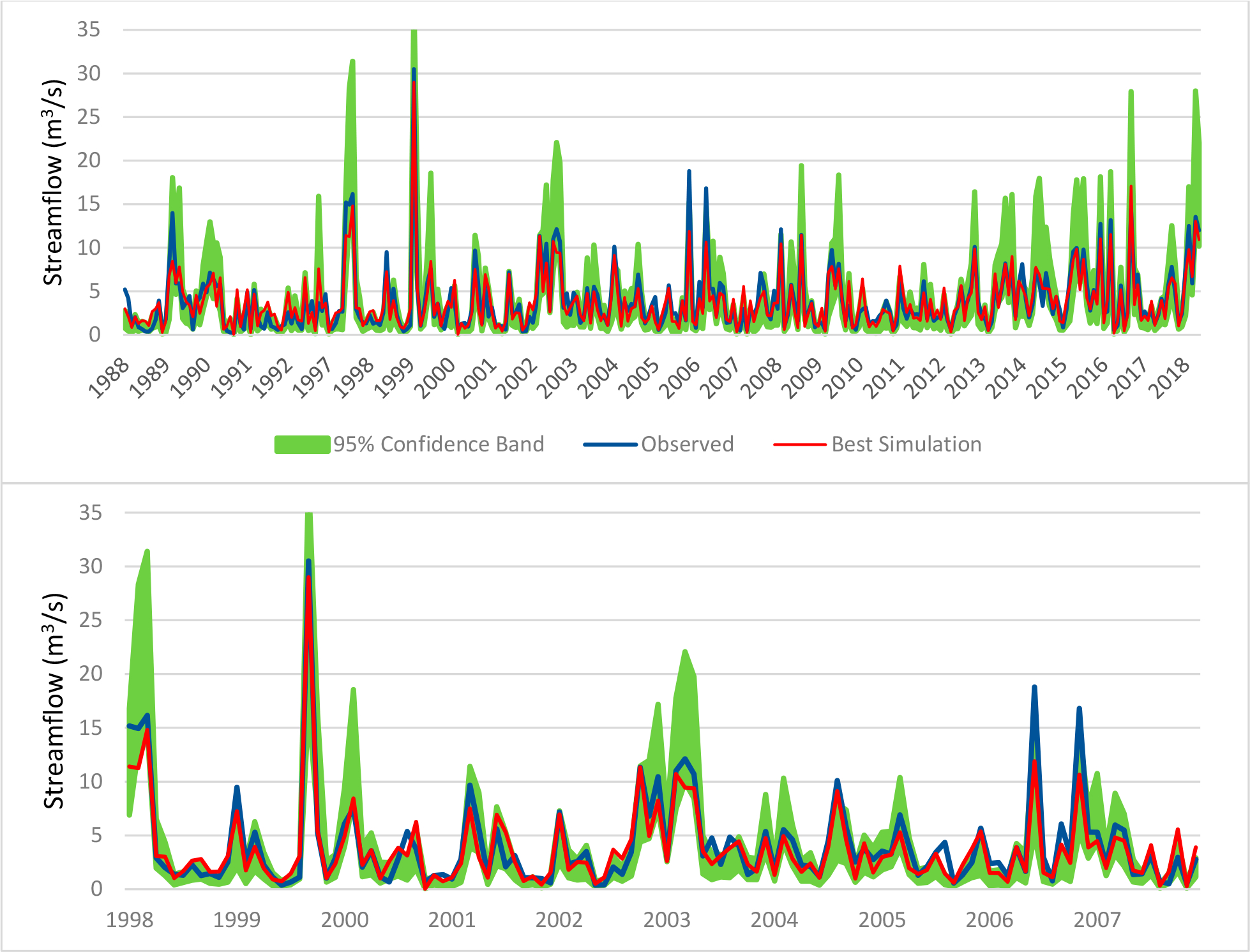
(**Top**) Sycamore Creek Monthly USGS (blue-observed) and SWAT simulated (best estimation) streamflow along with the 95% range for all simulations from 1988–2018. (**Bottom**) Zoomed-in to show detail for a 10-year period from 1998–2007.

**Figure A3.**
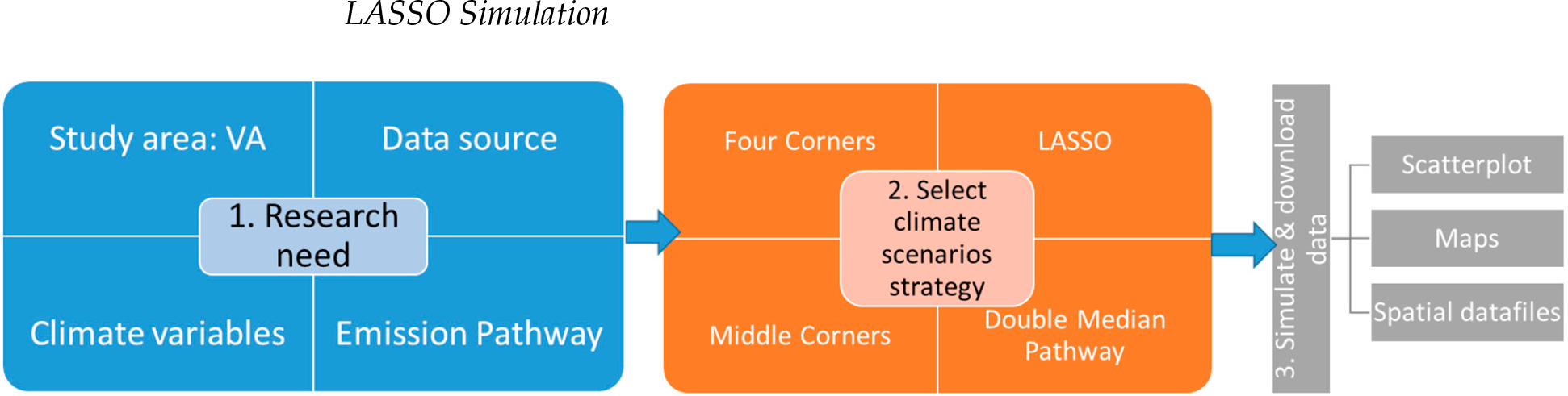
Locating and Selecting Scenarios Online (LASSO) systematic simulation approach for identifying a subset of future climate change projections of air temperature and precipitation. Note: climate variables include change in mean annual temperature (°C) and change in mean annual precipitation (%) for five times of year variables (annual, winter, spring, summer, fall) and three timeframe variables (2021–2050, 2041–2070, and 2070–2099). The emission pathway variables include two representative concentration pathways (RCPs), RCP4.5 and RCP8.5. The data sources are bias corrected spatially downscaled (BCSD) and localized constructed analogs (LOCA) datasets.

Through the HAWQS watershed calibration, we obtained a total of 13 acceptable parameter sets (Appendix A, Table A3) and used them to conduct subsequent HAWQS–RBZ and No-RBZ sensitivity modeling. We achieved HAWQS watershed model best performance statistics of KGE at 0.91 for Back Creek and the NSE at 0.87 for Sycamore Creek, reflecting the accuracy of the goodness of fit between the simulated and observed streamflow (Table 4, Figures A1 and A2). The value of NSE and KGE ranges from negative infinity to 1, where the value near 1 refers to a good fit of the model.

### 3.3. RBZ Sensitivity to WQI Parameters

**Figure 3.**
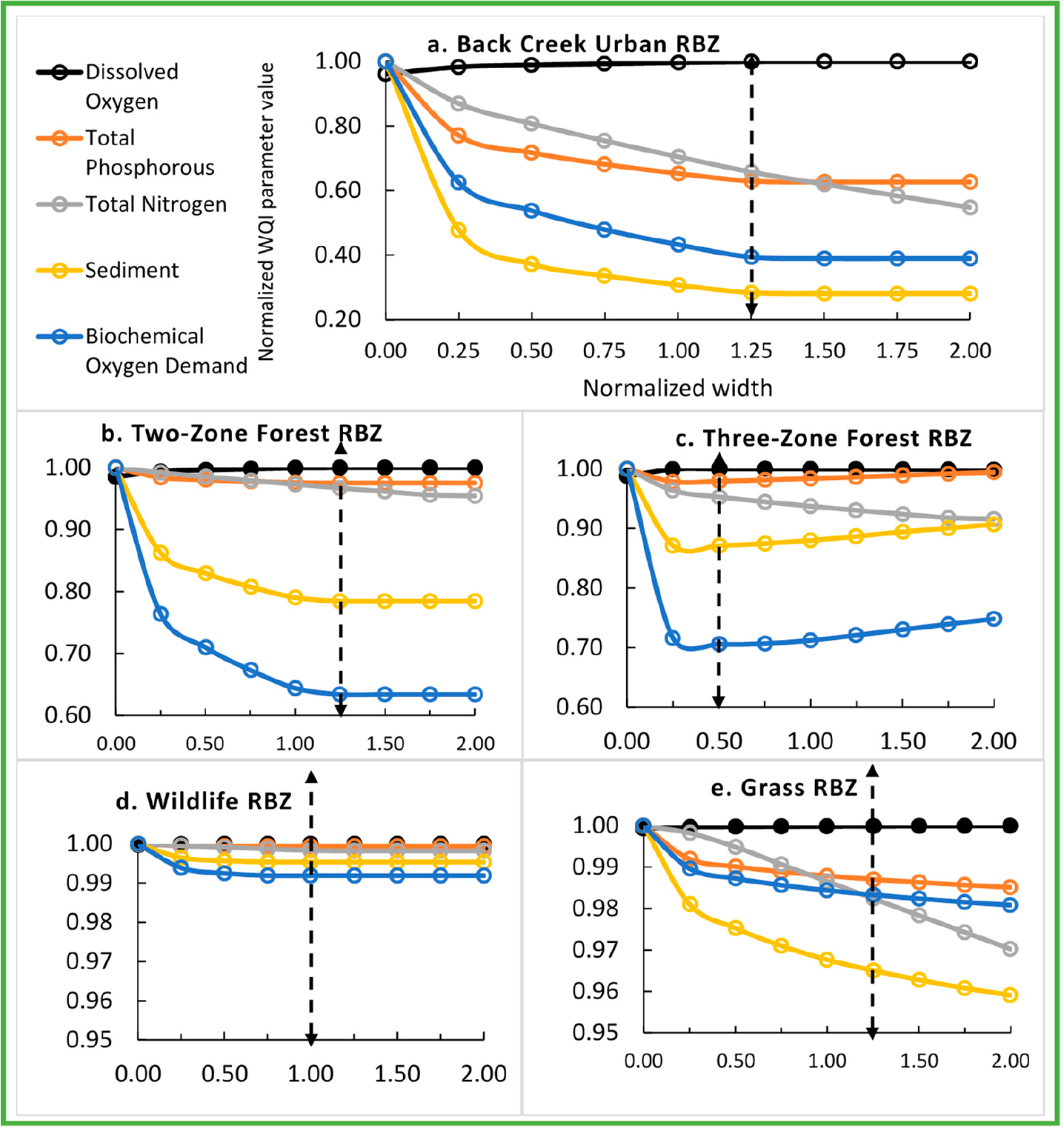
Sensitivity analyses revealing the optimal RBZ designs (dotted arrow) in Back Creek watershed. The dotted arrows indicate optimal width of urban, two-zone forest, three-zone forest, wildlife, and grass RBZs at 1.25, 1.25, 0.50, 1.00, and 1.25 times the baseline width, respectively.

The 36-y average daily WQI parameter concentrations under the baseline RBZs and No-RBZ are presented in Table 5. The WQI concentrations were highest in Greens Mill among the three watersheds except DO in Back Creek. Note that urban land use area dominated (70%) Greens Mill watershed. These concentrations were normalized with respect to maximum WQI parameter concentration, which corresponded to No-RBZ except for the DO. The baseline urban RBZ in Back Creek reduced SD, BD, TP, and TN by 69%, 57%, 35%, and 30%, respectively, and raised DO by 4% with respect to the No-RBZ (Figure 3).

**Figure 4.**
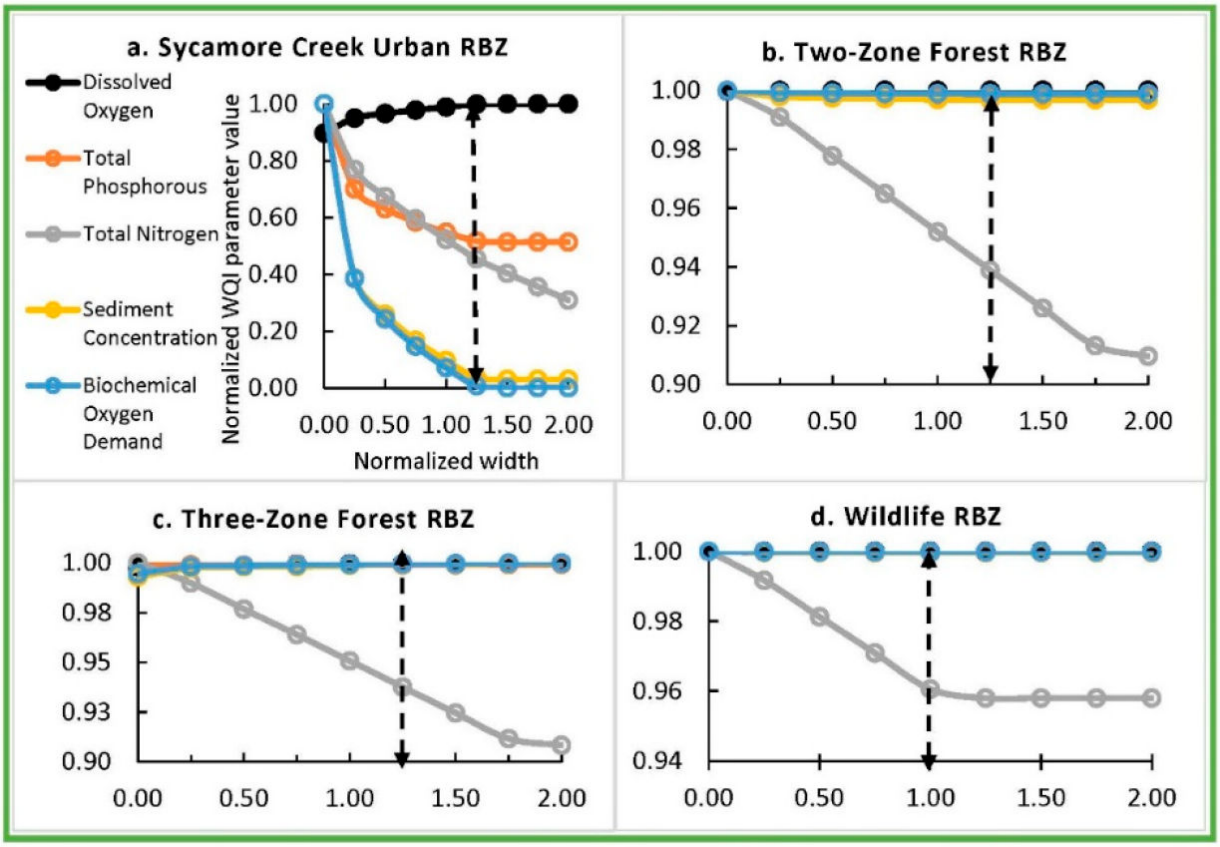
Sensitivity analyses revealing the optimal RBZ designs in Sycamore Creek watershed. The dotted arrows indicate optimal width of urban, two-zone forest, three-zone forest, and wildlife RBZs at 1.25, 1.25, 1.25, and 1.00 times the baseline width, respectively.

**Figure 5.**
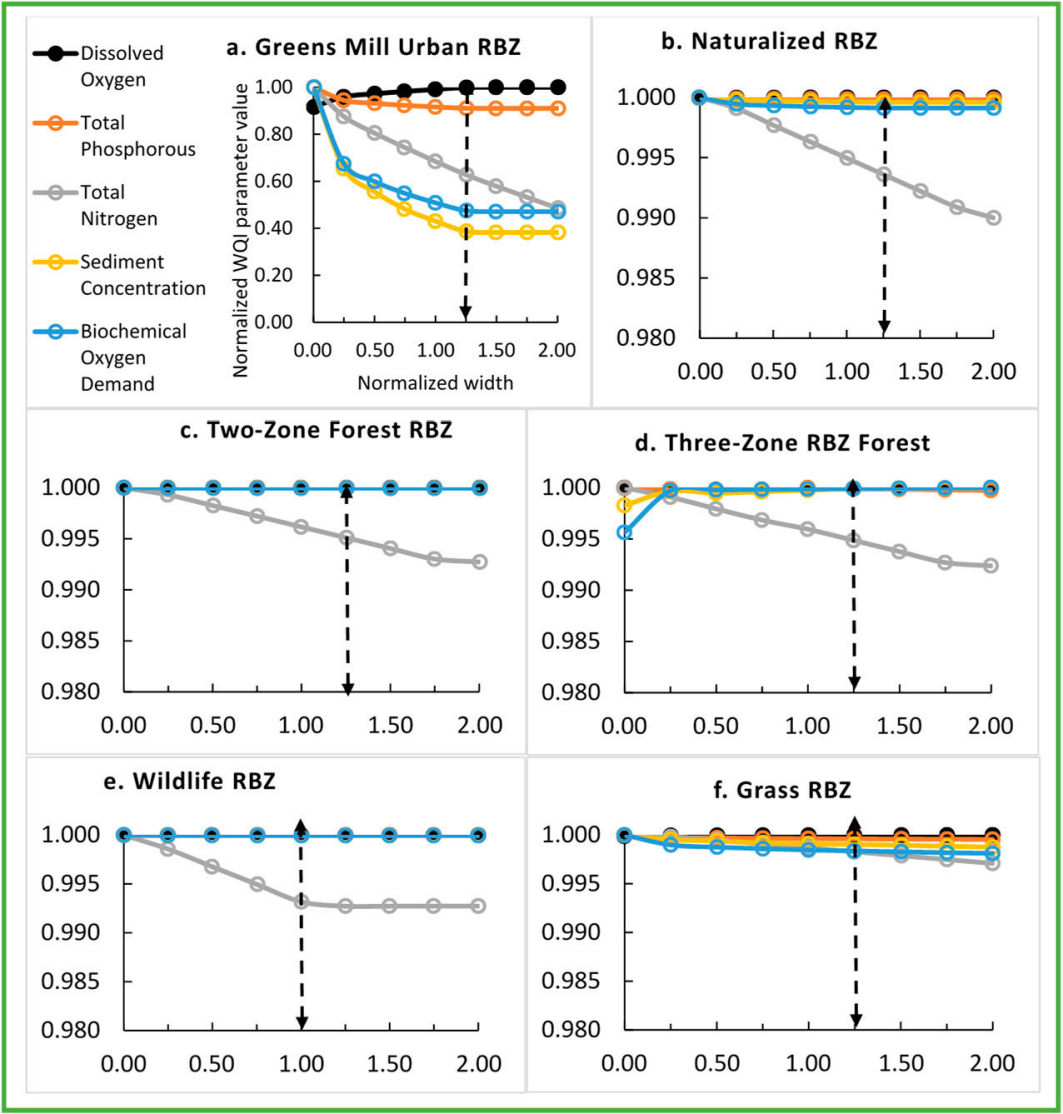
Sensitivity analyses revealing the optimal RBZ designs in Greens Mill Run watershed. The dotted arrows indicate optimal width of urban, naturalized, two-zone forest, three-zone forest, wildlife, and grass RBZs at 1.25, 1.25, 1.25, 1.25, 1.00, and 1.25 times the baseline width, respectively.

The baseline urban RBZ in Sycamore Creek reduced SD, BD, TP, and TN by 90, 93, 45, and 48%, respectively, and raised DO by 10% (Figure 4). The baseline urban RBZ in Greens Mill reduced SD, BD, TP, and TN by 57, 49, 8, and 32%, respectively, and raised DO by 8% (Figure 5). The sensitivity analyses revealed an optimal width for each RBZ design in each watershed. Optimal width, the dotted lines in Figures 3–5, corresponded to a width resulting in maximum potential improvements with an exception to TN due to urban RBZ. From Figures 3–5, it can be seen that the reduction in TN is very small (<3%) beyond the optimal width for all but the urban RBZ. The baseline RBZs (grass, Urban, two-zone forest, three-zone forest, wildlife, and naturalized RBZ) were 8, 23, 27, 34, 46, and 23 m wide, respectively. The optimal widths of urban, two-zone forest, three-zone forest, wildlife, and grass RBZs were found at 1.25, 1.25, 0.50, 1.00, and 1.25 times the baseline width, respectively, in Back Creek. In Sycamore Creek and Greens Mill, the optimal widths of all RBZs were found at 1.25 times the baseline width except the wildlife RBZ.

The percentage improvements were highest in Sycamore Creek due to optimal RBZ among the three watersheds. The optimal Urban RBZ in Back Creek, Sycamore Creek, and Greens Mill Run reduced SD by 72%, 96%, and 61%, respectively. The TN reductions due to the optimal urban RBZ in Back Creek and Greens Mill were similar (34% and 37%). The optimal urban RBZ raised DO by 4%, 10%, and 8%, respectively in Back Creek, Sycamore Creek, and Greens Mill Run.

### 3.4. LASSO—Climate Projections and Sensitivity to WQI Parameters

The scenario discovery approach resulted in 21 LASSO climate simulation options including an initial 15 options (i.e., 1 LOCA Data set × 1 RCP8.5 Pathway × 5 Seasons × 3 Time period × 1 LASSO Strategy = 15) and six additional options to justify the extremeness of the variables (Table A4). Finally, the systematic LASSO simulations resulted in four extreme climate models of annual changes in air temperature and precipitation through the LOCA dataset, the RCP8.5 Pathway, and the LASSO strategy for the late-century period of 2070–2099 (Table 6), to be integrated into the HAWQS–RBZ models.

Given the potential full range of plausible yet practically constrained future climate change models, our approach of choosing a subset of climate projections strikes a balance across scenarios by targeting extreme climates and selecting relevant climate information. In Back Creek, the extreme average annual precipitation changed from −3.2% (Dry) to 23% (Wet), which were higher than in Sycamore Creek and Greens Mill (changing from −10% (Dry) to 19.5% (Wet)) during the late century (2070–2099). The extreme temperatures in Back Creek were projected to increase from 2.7 to 6.3 °C, which were more extreme than in Sycamore and Greens Mill (Table 6).

**Figure 6.**
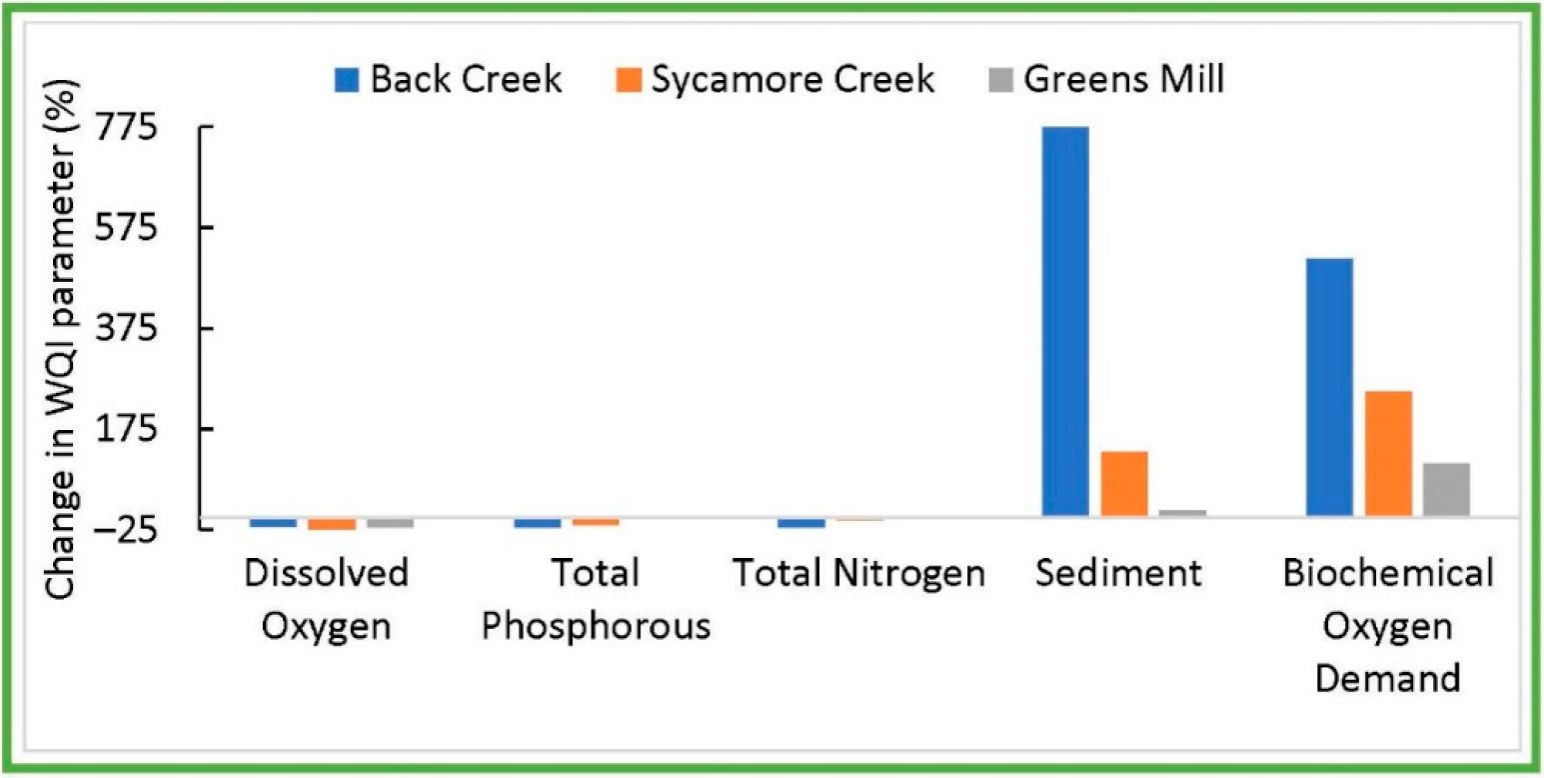
The impacts of the future climate change (the late century’s (2070–2099) extreme annual changes in air temperature and precipitation) on water quality indicator (WQI) parameters in three watersheds. The contemporary climate No-RBZ scenario is compared with the future extreme climate No-RBZ.

**Figure A4.**
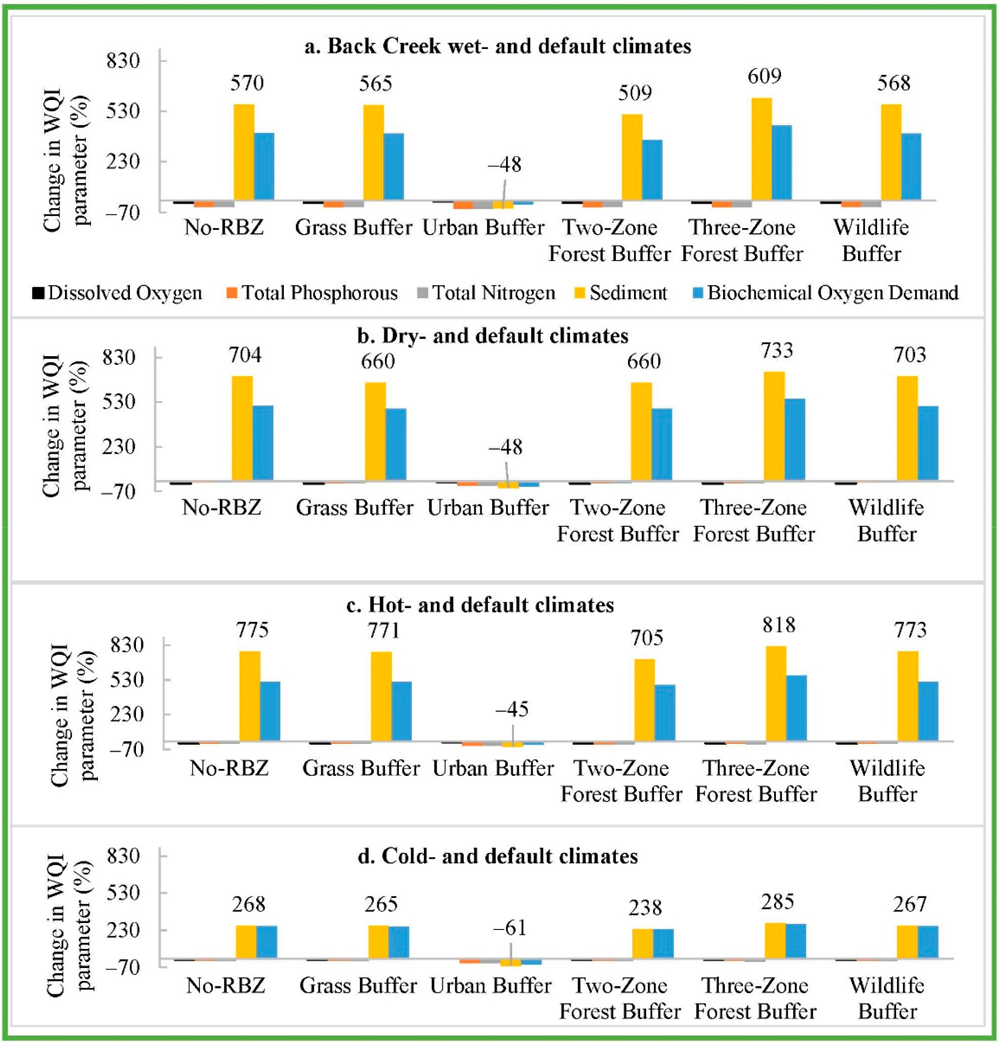
Back Creek future climate-baseline RBZ impacts compared with contemporary climate No-RBZ impacts.

**Figure A5.**
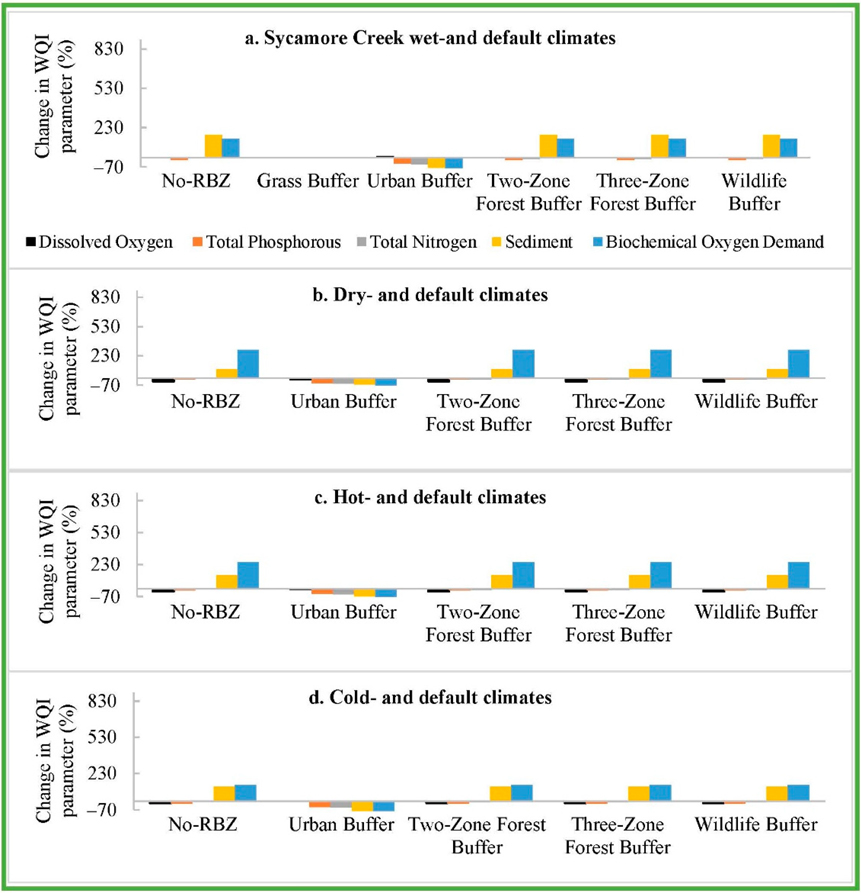
Sycamore Creek future climate-baseline RBZ impacts compared with contemporary climate No-RBZ impacts.

**Figure A6.**
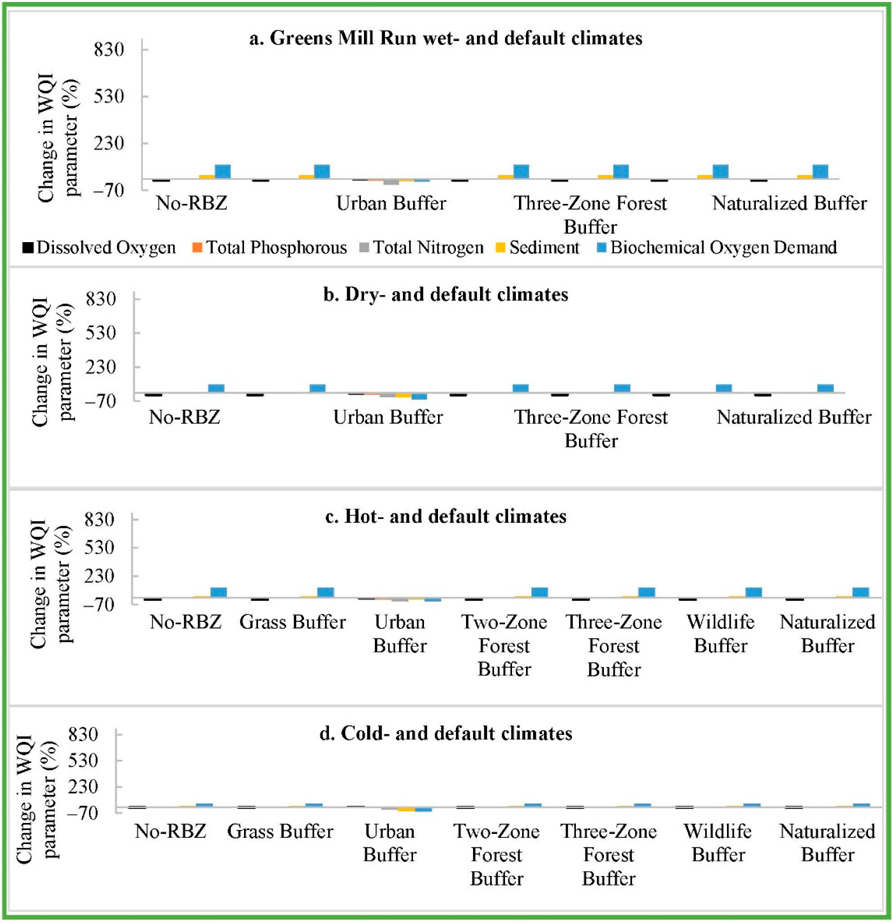
Greens Mill Run future climate-baseline RBZ impacts compared with contemporary climate No-RBZ impacts.

Integration of the extreme temperature and precipitation into the HAWQS–RBZ models resulted in watershed-specific results. The late-century climate change conditions increased the projected SD and BD by an average of 775% and 512% with respect to contemporary climate No-RBZ (Figure 6, also see Appendix A Figures A4–A6).

**Figure 7.**
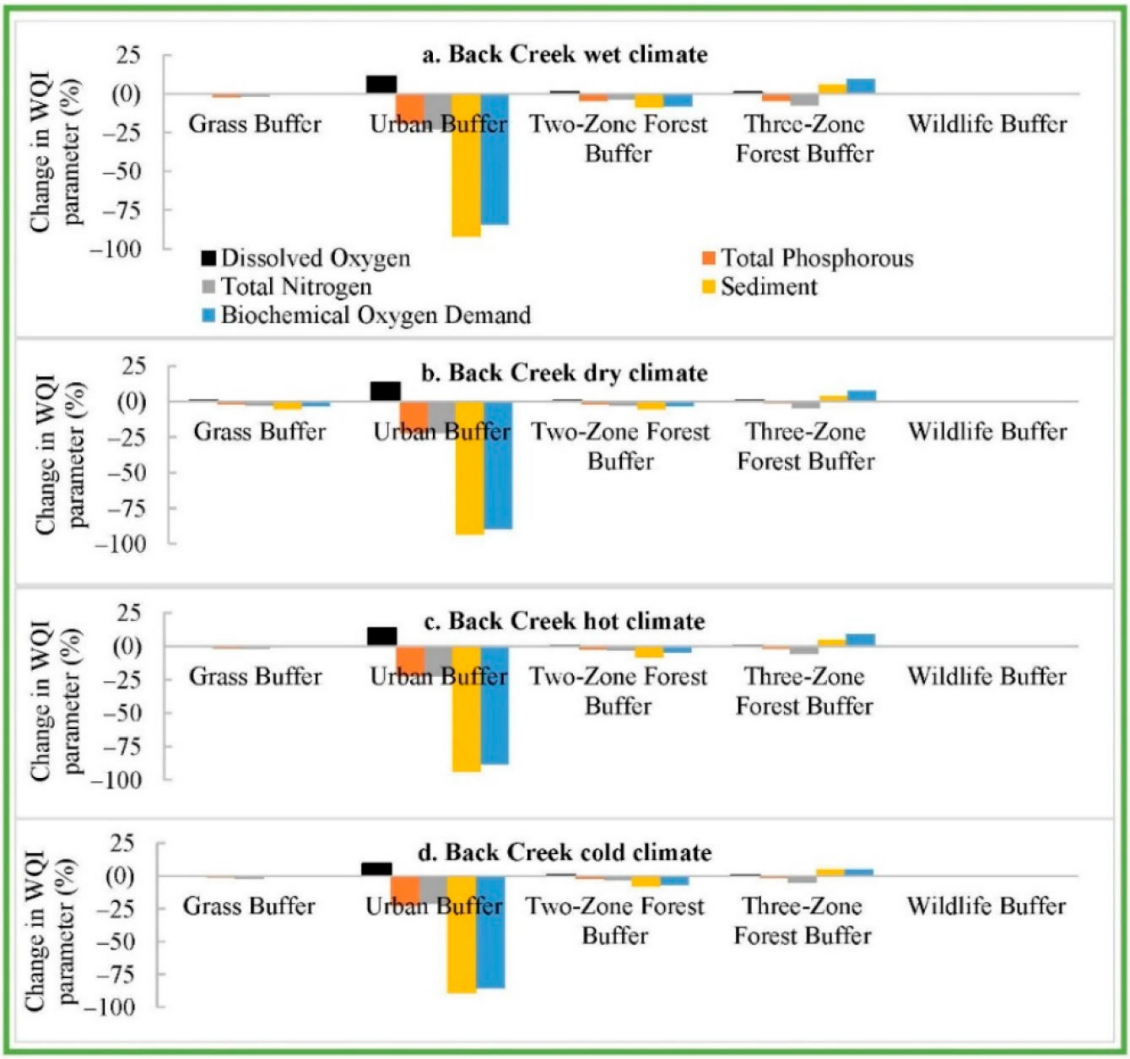
Back Creek future climate-baseline RBZ impacts compared with future climate No-RBZ impacts.

**Figure 8.**
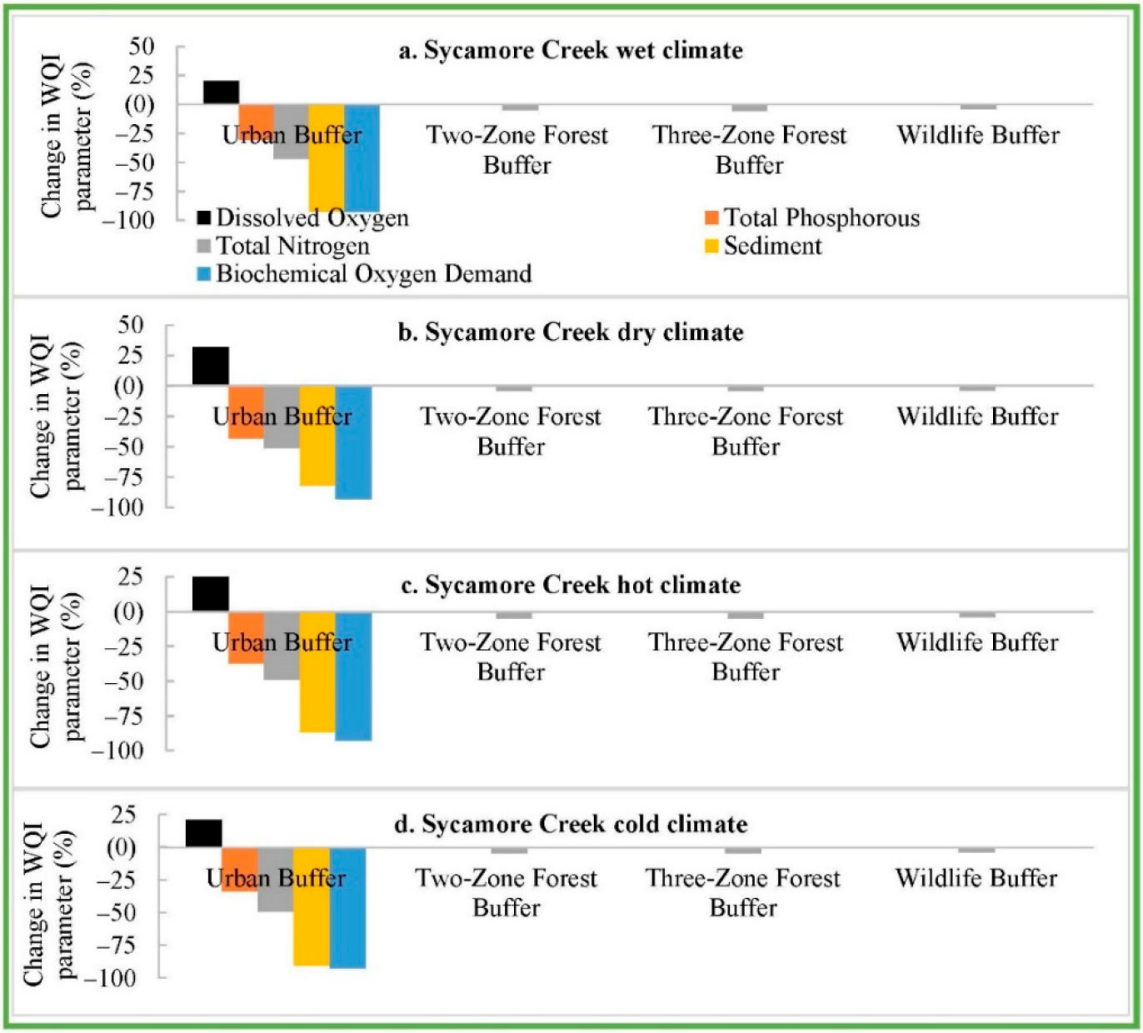
Sycamore Creek future climate-baseline RBZ impacts compared with future climate No-RBZ impacts.

**Figure 9.**
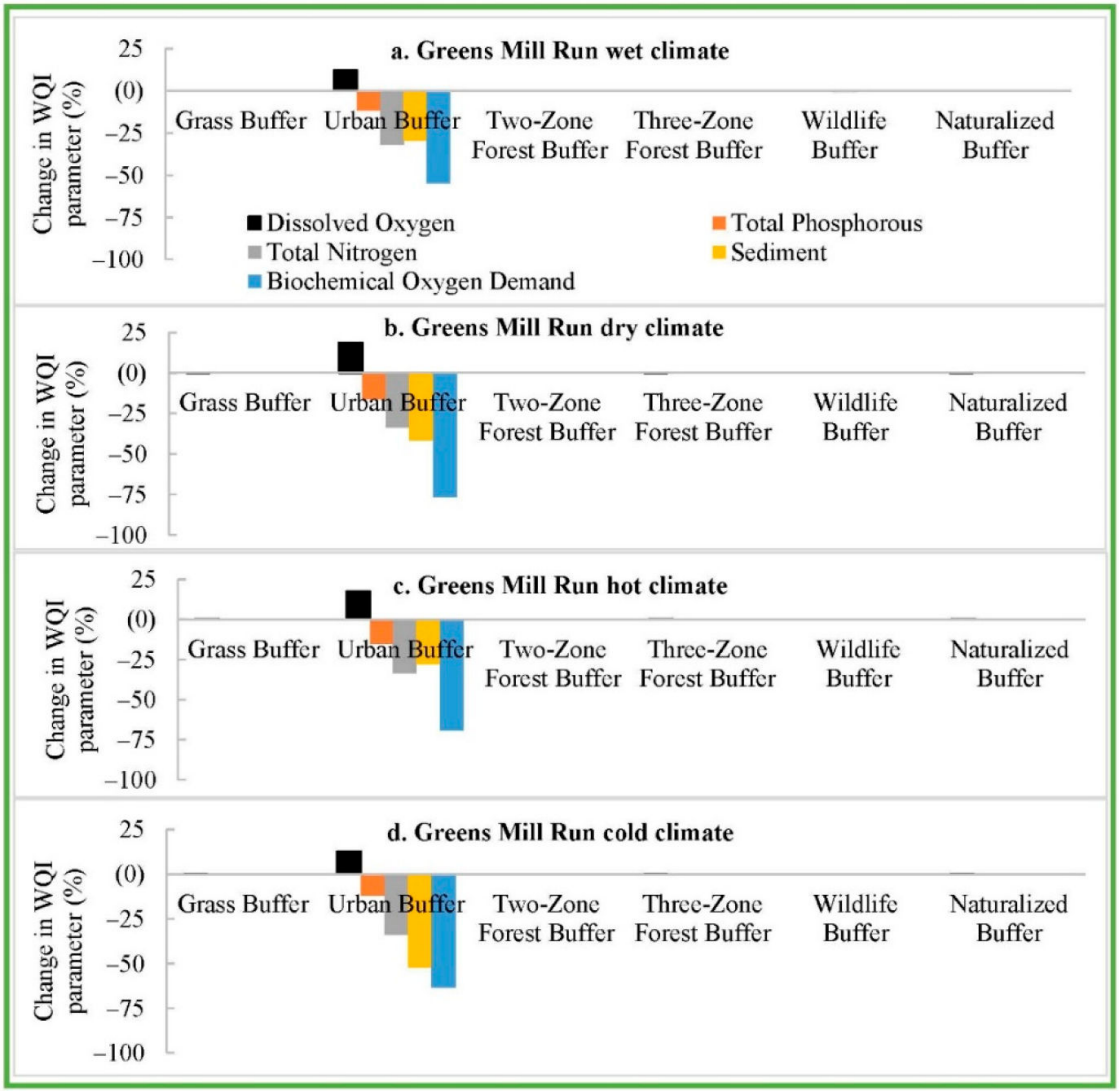
Greens Mill Run future climate-baseline RBZ impacts compared with future climate No-RBZ impacts.

The future climate RBZ impacts (except the Urban RBZ impacts), compared with the contemporary climate No-RBZ impacts, also showed significant increase in SD and BD as provided in (Appendix A Figures A4–A6). However, the addition of urban RBZ to future climate No-RBZ baseline resulted in a projected reduction in SD and BD by 94% and 88% in Back Creek (Figure 7). The addition of urban RBZ in Sycamore Creek also reduced SD and BD by 87% and 93% (Figure 8), with lower reductions (28% and 69%) in Greens Mill watershed (Figure 9). All other types of RBZs are also projected to fully offset the climate change impacts on WQI parameters except three-zone RBZ (Figures 7–9).

## 4. Discussion

The study suggested watershed-specific yet generalizable water quality implications of RBZ design scenarios:

Watershed-specific RBZ-WQI tradeoffs support RBZ management decisions. The WQI parameter results provided valuable information for selecting a preferred RBZ width guided by the total maximum daily load (TMDL), a specified maximum amount of a pollutant allowed to a waterbody under applicable water quality standards [33]. For example, the optimal urban RBZ in contemporary climate conditions in Back Creek reduced SD, BD, TP, and TN by 72%, 61%, 37%, 34%, respectively, and raised DO by 4% with respect to the maximum value of No-RBZ. If the goal was to reduce TN loads from agricultural areas with no limitations on resources (e.g., land use and RBZ implementation costs), a wider urban RBZ (twice the baseline width) would be implemented in which TN could be further reduced to 15%, 14%, and 11% in Sycamore Creek, Greens Mill, and Back Creek watersheds, respectively. Wider RBZs would lower WQI parameters in addition to protecting the wildlife habitat. Oftentimes, the primary goal is to improve the presence of DO, which is critical in maintaining aquatic life. To put this into perspective, the North Carolina Department of Environment, Health, and Natural Resources specified water quality standards for DO at a daily average of 5.0 mg/L or greater for all surface waters, and not less than a minimum instantaneous value of 4.0 mg/L for fresh waters (as per the NC Administrative Code:15A NCAC 2B 0.0100 and SA NCAC 2B 0.0200) [60] in the Albemarle-Pamlico estuary area. The Sycamore Creek’s simulated DO level at 3.7 mg/L (Table 4) was below the recommended average values for all surface waters yet the optimal urban RBZ raised the DO level (10% increase from the baseline, Figure 4), which was still lower than the recommended concentration. The DO concentrations in Back Creek and Greens Mill Run were greater than the recommended level, however. The simulated BD concentrations in all watersheds were below the water quality standard value (5.0 mg/L) at or above which the BD parameter would be considered high, indicating the presence of a source of organic matter [60].

The optimal RBZs offer maximum WQI benefits; however, there is no “one-size-fits-all” design of an ideal RBZ [13]. We recommended optimal widths for each of the six baseline RBZ designs. Wider widths may be considered to protect streams from a specific nutrient, for streambank stabilization, or for provision of wildlife habitat. The width adjustment may occur due to landowner interests in land, money (e.g., cost-share and/or tax-incentive programs), the adoption of a specific type of RBZ, or RBZ management efforts, such as a frequent timber harvest to maintain active pollutants sequestering and a deep and fibrous root system to increase soil quality parameters (organic matter, microbial functional diversity, and enzyme activities) [61]. In fact, a study suggested that the RBZ adoption rate would likely increase if incentive programs allowed for more flexible buffer designs [62].

The simulated RBZs were favorable to serve as a climate adaptation strategy in the face of projected extreme climate conditions. Although future extreme climate increased SD (from 15–775%) and BD (from 107–512%) compared to the current condition of No-RBZ in the late century (2070–2099), the application of all but the three-zone forest RBZs are projected to fully offset or lower the climate change WQI parameters in all watersheds. The projected changes in extreme air temperatures (6.3 °C in VA and 5.7 °C in NC) were greater than projected extreme surface temperature increases (2.6–4.8 °C in late 21st century, projected by the Intergovernmental Panel on Climate Change (IPCC)) [10]. The extreme temperature was found to be a stronger driver of stream water quality change compared to precipitation, potentially resulting from the loss of snow cover in the late winter months that increased water erosion.

The WQI-tradeoffs were watershed-specific, suggesting a watershed-specific assessment. Current study showed watershed-specific results with reductions in TN from 34–55%, TP from 37–48%, and SD from 72–96% due to the optimal urban RBZ. Yet, these results were similar to past studies that suggested a wide variation in the RBZ’s effectiveness. For example, a global study reported nitrogen removal efficiency from 20–100% [14]; total phosphorous removal efficiency was reported from 27–97% in Finland, Norway, Sweden, and Denmark [23]; sediment trapping was reported from 84–90% in North Carolina [25]; orchard grass filter strips removed 84% of sediment and soluble solids from surface runoff in Virginia [28].

These water quality implications of RBZ scenarios can provide valuable insights for making informed RBZ decisions to protect and restore stream water quality under 303(d) of the Clean Water Act [33] in the face of future climate change. As an example, our methods can be useful for assessing the effectiveness of existing programs, such as the Albemarle-Pamlico National Estuary Partnership’s (APNEP) Comprehensive Conservation and Management Plan (2012–2022)—a jointly sponsored program to protect and restore the significant resources of the estuarine system by the NC Department of Environment and Natural Resources and the Virginia Department of Conservation and Recreation with financial support from EPA [63]. Nationwide, the methods can be supportive to the USDA’s Conservation Reserve Enhancement Program (CREP), which promotes the development of riparian buffers along streams [64].

Addressing study limitations and going forward. The results reflected the RBZ tradeoffs in headwater streams (i.e., first, second, and third order streams), settings that can have greater influences on overall water quality impacts than those occurring in downstream higher order reaches [65]. In the higher order reaches, the RBZs tend to be wider, providing a significant wildlife habitat. The variation in average RBZ width (±100% variation) addressed the adjustable nature of the width. It is noted that the fixed-width RBZ recommendations tend to be easier to enforce and administer by regulatory agencies; however, the fixed-width often fails to provide for a variety of ecological functions compared to the adjustable RBZ width, which is generally adjusted along the length of the RBZ depending on adjacent land use, site conditions (e.g., vegetation, topography, hydrology), fish and wildlife considerations [13], and most importantly, stream water conditions and TMDL goals.

Future research should focus on RBZ designs and holistic watershed management through a cross-disciplinary approach to eco-efficiency and sustainability [66]. The cross-disciplinary approach may begin with RBZ cost estimation; quantification and monetization of the benefit-transfer of water quality changes; assessment of the potential multifunctional ecosystem services—the environmental, economic, and social benefits of RBZ. We addressed water quality indicators as a component of the environmental aspect; however, future efforts should also consider assessing RBZ’s holistic impacts on “one water” (all types of waters receiving the non-point source pollutants) [67] resources at the watershed scale. The economic aspect may include the RBZ costs (implementation, management, and agricultural and timber opportunity costs), as well as land rental and maintenance payments through the Continuous Conservation Reserve Program (CCRP) [64]; the social aspects may include physical and emotional well-being as a result of increased living standard and income from RBZ. Additional research consideration towards holistic watershed management may include an assessment of watershed proper functioning conditions (hydrology, vegetation, and topography/soils) [20] and both hydrology (flow) and water quality calibration, including sediment, total nitrogen, total phosphorous, and dissolved oxygen depending on the availability of observed data.

## 5. Conclusions

We formulated 135 RBZ design scenarios and conducted sensitivity analyses of WQI parameters in three HUC-12 watersheds within the Albemarle-Pamlico river basin. We discussed generalizable study implications of RBZ-WQI tradeoffs in the contemporary climate and in future late-century extreme climatic conditions. The study intended to support RBZ decisions to protect stream water quality and restore impaired waters under 303(d) of the CWA that also authorizes EPA to assist states, territories, and tribes in developing TMDLs. Key study implications are summarized below:

We summarized the RBZ’s design strategy and evaluated RBZ water quality indicator (WQI) parameters as a component of watershed ecosystem services at the watershed scale. The analyses revealed optimal widths that corresponded to 1.25 times the baseline width of the RBZ designs modeled in all of the three watersheds, except the wildlife and three-zone forest RBZs. Urban RBZs were found to be the most sensitive of all watersheds. There is no “one-size-fits-all” design for an ideal RBZ [13]; therefore, the RBZ-WQI tradeoffs analyses are recommended to inform the width selection according to TMDL goals, for streambank stabilization, or for provision of wildlife habitat.

- The WQI parameter tradeoffs were watershed-specific and influenced by future extreme climates, suggesting a watershed-specific assessment.
- In terms of watershed ecosystem services, the optimal urban RBZ under contemporary climate reduced TP, TN, SD, and BD by 48%, 55%, 96%, and 99%, respectively, and raised dissolved oxygen by 10% with respect to the maximum values of No-RBZ in Sycamore Creek. The projected future extreme climate change significantly increased the projected SD and BD with respect to the current climate no-RBZ condition in Back Creek; however, the addition of an Urban RBZ resulted in reductions of SD and BD by 94% and 88%.
- Current models are transferrable to simulate RBZs along hydrologically connected aquatic systems of rivers, lakes, ponds, and wetlands in the U.S., from eastern to southeastern to the midwestern regions, yielding average to substantial quantities of non-point source pollutants into surface and ground waters by obtaining watershed-specific information, such as geographic database and spatial/temporal weather data. Current methods and findings are useful in assessing the effectiveness of RBZ policies in the Southeast U.S. and outside with similar watershed characteristics. For example, the methods can be useful for developing RBZ design policies to assess the effectiveness of the Comprehensive Conservation and Management Plan (2012–2022) of the Albemarle-Pamlico National Estuary Partnership (APNEP) [63]. Nationally, the outcomes can support the U.S. government’s various riparian restoration and preservation programs, including the USDA Conservation Reserve Program, which promotes the development of riparian buffers along streams [64].
- This study serves as the first step towards RBZ designs and holistic watershed management through a cross-disciplinary approach of eco-efficiency and sustainability [66].

## Figures and Tables

**Table 1.** Baseline (starting point) riparian buffer zone (RBZ) designs for sensitivity analyses [41]. The average width measures (rounded to the nearest meter) are for one side of the stream.

RBZ Type	Average Width (m)	Description
Grass Buffer	8	This buffer consists only of grasses and forbs and is typically used along small streams and other drainages that flow through crop fields and pastures. The literature suggested buffer width is 6.1 m to 9.1 m.
Three-Zone Forest Buffer	34	This buffer consists of three zones: zone 1 (undisturbed forest), ranging in width from 4.6–9.1 m, contains trees along the edge of the stream; zone 2 (managed forest), ranging in width from 9.1–30.5 m, filters sediment that passes through zone 3 and absorbs nutrients while providing wildlife habitat; zone 3 (runoff control), 6.1–9.1 m wide, is usually a grass strip. The literature suggested that the minimum total buffer width on each side of a stream is 15.2–30.5 m but should be wider with increasing slope.
Two-Zone Forest Buffer	27	A two-zone forest buffer would simply be a modification to the three-zone forest buffer, where the grass zone (zone 3) would not be established.
Urban Buffer	23	This buffer consists of low-, medium-, and high-density residential land use types. The buffers can be used to teach users (homeowners and developers) about the RBZ’s water quality benefits. The literature suggested buffer width is 15.2–30.5 m.
Wildlife Buffer	46	This buffer consists of evergreen forest. A wildlife buffer is usually wider, to better function as a travel corridor and connector between larger tracts of forest. Suggested buffer width is up to 91.4 m.
Naturalized Buffer	23	This buffer consists of forested wetlands. This is an inexpensive natural buffer that can still effectively intercept runoff. Existing vegetation can be supplemented by interplanting tree and shrub seedlings. The literature suggested buffer width is 15.2–30.5 m.

**Table 2.** Conceptual matrix of 135 sensitivity analysis design scenarios and the RBZ width in meters (rounded to the nearest meter). All six baseline RBZ vegetations and nine width variation designs were applied to Greens Mill Run watershed resulting into 54 scenarios (i.e., 6 × 9 = 54). Five of the six baseline designs were applied to Back Creek, with the exception of naturalized RBZ (i.e., 5 × 9 = 45). Four of the six baseline designs were applied to Sycamore Creek, with the exception of grass and naturalized RBZs (i.e., 4 × 9 = 36) due to land use type limitations.

RBZ Width Variation Scenario (S*_i_*) (*i* = 0 to 8)	RBZ Vegetation (V*_i_*)					
	Grass Buffer	Urban Buffer	Two-Zone Forest Buffer	Three-Zone Forest Buffer	Wildlife Buffer	Naturalized Buffer

0.0 × baseline width (S0) (No-RBZ)	0	0	0	0	0	0
0.25 × baseline width (S1)	2	6	7	9	11	6
0.50 × baseline width (S2)	4	11	13	17	23	11
0.75 × baseline width (S3)	6	17	20	26	34	17
Baseline (1.0) RBZ width (S4)	8	23	27	34	46	23
1.25 × baseline width (S5)	10	29	33	43	57	29
1.50 × baseline width (S6)	11	34	40	51	69	34
1.75 × baseline width (S7)	13	40	47	60	80	40
2.0 × baseline width (S8)	15	46	53	69	91	46

**Table 3.** Watershed properties and land use defining the riparian buffer zones (RBZs) (The Table was modified with permission from Ghimire and Johnston 2013 [35], Copyright Elsevier (2013)).

Watershed	Stream Length (km)	Area (km^2^)	Population Density (People/km^2^)	Elevation Range (m)	Precipitation (cm)	Urban RBZ	Grass RBZ	Two-Zone Forest RBZ	* Three-Zone Forest RBZ	Wildlife RBZ	Naturalized RBZ

Back Creek	40.0	152	100	305–646	106	LMDR (19%)	Hay (7%)	EF/DF (74%)	EF/DF (74%)	EF (4%)	NA
Sycamore Creek	12.1	41.7	600	74–136	111	LMHDR (51%)	NA	EF/DF (49%)	EF/DF (49%)	EF (23%)	NA
Greens Mill Run	22.9	34.7	900	0–46	127	LMHDR (70%)	Range-Brush (4%)	EF (8%)	EF (8%)	EF (8%)	FW (13%)

Note: LMDR = low-, medium-density residential; LMHDR = low-, medium-, high-density residential; EF/DF = evergreen forest/deciduous forest; FW = forested wetlands; NA = not available

* EF was used as proxy for grass RBZ for the purpose of three-zone Forest simulation in Sycamore Creek due to land use type limitations.

**Table 4.** A summary of the Nash and Sutcliffe efficiency (NSE), Kling–Gupta Efficiency (KGE), and percent Bias (PBIAS) as model performance statistics for the watershed models.

Watershed	PBIAS	NSE	KGE	Average Flow (Observed) (m^3^/s)	Calibration Period
Back Creek	−3.90%	0.83	0.91	1.92 (1.85)	1983–2018
Crabtree Creek (Sycamore Creek)	3.80%	0.87	0.85	3.75 (3.9)	1988–2018

**Table 5.** Water quality indicator (WQI) parameter concentrations in three watersheds with baseline riparian buffer zones (RBZs) and without RBZs (i.e., No-RBZs). The values are 36-year average daily concentrations from 1983–2018.

**Back Creek Watershed Baseline RBZs: All Values in mg/L**							
**Water Quality Indicator Parameter**	**No-RBZ**	**Grass Buffer**	**Urban Buffer**	**Two-Zone Forest Buffer**	**Three-Zone Forest Buffer**	**Wildlife Buffer**	**Naturalized Buffer**
Dissolved Oxygen	9.6	9.6	10.0	9.7	9.7	9.6	NA
Total Phosphorous	0.2	0.2	0.1	0.2	0.2	0.2	NA
Total Nitrogen	2.0	2.0	1.4	2.0	1.9	2.0	NA
Sediment Concentration	12.7	12.3	3.9	10.1	11.2	12.7	NA
Biochemical Oxygen Demand	0.5	0.5	0.2	0.3	0.3	0.5	NA
Sycamore Creek Watershed Baseline RBZs: All values in mg/L							
Water Quality Indicator Parameter	No-RBZ	Grass Buffer	Urban Buffer	Two-Zone Forest Buffer	Three-Zone Forest Buffer	Wildlife Buffer	Naturalized Buffer
Dissolved Oxygen	3.7	NA	4.1	3.7	3.7	3.7	NA
Total Phosphorous	0.2	NA	0.1	0.2	0.2	0.2	NA
Total Nitrogen	1.3	NA	0.7	1.3	1.3	1.3	NA
Sediment Concentration	19.3	NA	1.9	19.3	19.4	19.3	NA
Biochemical Oxygen Demand	0.5	NA	0.0	0.5	0.5	0.5	NA
Greens Mill Run Watershed Baseline RBZs: All values in mg/L							
Water Quality Indicator Parameter	No-RBZ	Grass Buffer	Urban Buffer	Two-Zone Forest Buffer	Three-Zone Forest Buffer	Wildlife Buffer	Naturalized Buffer
Dissolved Oxygen	6.8	6.8	7.4	6.8	6.8	6.8	6.8
Total Phosphorous	0.4	0.4	0.4	0.4	0.4	0.4	0.4
Total Nitrogen	3.3	3.3	2.2	3.3	3.3	3.3	3.3
Sediment Concentration	61.0	60.9	26.2	61.0	61.0	61.0	60.9
Biochemical Oxygen Demand	2.2	2.2	1.1	2.2	2.2	2.2	2.2

Note: NA = not available.

**Table 6.** LASSO future climate projection results for the states of VA and NC.

Climate Code	Climate Projection Scenario Name	Virginia		North Carolina	
		Annual Precipitation Change, ΔP (%)	Annual Air Temperature Change, ΔT (°C)	Δ P (%)	Δ T (°C)
Hot	Hadley Centre Global Environment Model version 2-Carbon Cycle (HadGEM2-CC)	6.8	6.3	5.1	5.7
Cold	Russian Institute for Numerical Mathematics Climate Model Version 4 (inmcm4)	−0.2	2.7	−1.3	2.4
Dry	Flexible Global Ocean–Atmosphere–Land System Model, Grid-point Version 2 (FGOALS-g2)	−3.2	5.4	−10	5
Wet	Max Planck Institute for Meteorology Earth System Model Mix Resolution (MPI-ESM-MR)	23	4	19.5	3.8

## Data Availability

The data presented in this study are openly available within the manuscript and its Appendix A files. Additional data are published through the EPA ScienceHub (https://www.data.gov/).

## Appendix A

**Table A1.** Riparian buffer zone (RBZ) design literature review summary (Google Scholar Search criteria = allintitle: “riparian buffer design”). Two duplicates, four unrelated to RBZ designs, and three non-English text-literature were excluded.

Author (Year)	Riparian Buffer Designs	Summary
Jiang, et al. [68]	Proposed six RBZ designs: (i) 10 m grass, (ii) 15 m grass, (iii) 15 m deciduous trees, (iv) 30 m grass and trees, (v) 30 m grass and trees with trees harvested every 3 years, and (vi) 30 m grass and trees with grass harvested every year.	Evaluated watershed scale nutrient and sediment loads using soil and water assessment tool (SWAT) coupled with the riparian ecosystem management model (REMM). Reported reduction efficiencies of 76–78% for total nitrogen, 51–55% total phosphorus, and 68% sediment. Recommended flexible, farmer-preferred buffer design policies.
Jiang, et al. [62]	Proposed six RBZ designs: Design 1 (10 m zone 1 grass); Design 2 (15 m zone 1 grass); Design 3 (15 m zone 1 deciduous trees); Design 4 (6 m zone 1 grass, 19 m zone 2 desiduous trees, and 5 m zone 3 deciduous trees); Design 5 (6 m zone 1 grass, 19 m zone 2 deciduous trees harvested every 3 years, and 5 m zone 3 deciduous Trees); Design 6 (6 m zone 1 grass harvested every 3 years, 19 m zone 2 deciduous trees, and 5 m zone 3 deciduous trees).	Three crop rotations were also simulated using the SWAT coupled to the REMM. Reported annual total reduction rate of sediments and dissolved N at 90.8% and 91.9%.
Piscoya, et al. [69]	Reported a range of RBZ width from 7.6 m to 15.3 m for a small (2.10 km^2^) semiarid Brazilian watershed.	Determined RBZ width using a variable width equation for the incoming discharges, sediment loads, the slope, area, and annual soil loss. Concluded that the sediment yield time and hydrological data were important factors for determining the RBZ width.
Liu, et al. [70]	Created five RBZ designs: (i) Design 1 (10 m zone 1 of non-harvestable deciduous upper canopy forest next to the river + 20 m zone 2 of harvestable deciduous upper canopy forests in the middle + 20 m zone 3 of herbaceous perennials next to the field); (ii) Design 2 (10 m zone 1, 20 m zone 2); (iii) Design 3 (10 m zone 1); Design 4 (10 m zone 1, 10 m Zone 2); Design 5 (10 m zone 3 and 10 m zone 1).	Assessed impact of designs on water quality in a watershed in China using the Agricultural Non-Point Source Pollution Model (AnnAGNPS) and the REMM. Reported the removal efficiency of sediments from 85.7% to 90.8% and dissolved nitrogen in surface runoff from 85.4% to 91.9%.
Santin, et al. [71]	Reported the largest width value at 47 m and suggested the vegetation cover type as the single most relevant variable among soil type, mean nitrogen influent, and nitrogen removal effectiveness.	Proposed a method to estimate the riparian buffer width (the output) as a function of vegetation cover type, soil type, mean nitrogen influent, and nitrogen removal effectiveness (the inputs) in an agricultural watershed in Brazil using artificial neural networks (ANNs) and literature data.
Tomer, et al. [72]	Classified riparian sites into three groups according to runoff-contributing areas (the ratio of contributing area to buffer area): (i) high (potential to receive overland flow from large upslope areas); (ii) medium (wider than 10 m to provide a buffer-contributing area ratio of 0.02); (iii) low (narrow buffer, 10-m wide or less, provides the minimum recommended contributing area ratio of 0.02).	Proposed a geographic information system or GIS-based approach to identify riparian management alternatives in Iowa and Illinois using light detection and ranging (LiDAR) with high-resolution digital elevation models (DEMs).
Cardinali, et al. [73]	Compared four buffer designs in an experimental crop field of 200 m × 35 m: (i) 3 m wide grass buffer; (ii) 3 m grass with one tree row; (iii) 6 m grass with one tree row; (iv) 6 m grass with two tree rows in an experimental farm in Italy	Evaluated the buffer impacts on soil quality parameters (soil organic matter, soil microbial functional diversity, and soil enzyme activities). Reported that the 3 m grass and 3 m grass with one tree row buffers produced the highest values of soil organic matter quality parameters.
Johnson and Buffler [74]	Presented unadjusted optimal buffer widths for four slope categories and three hydrologic soil groups—from 21 m (70 ft) for the lowest slope range of 0% to 5% and high surface roughness hydrologic group to 58 m (190 ft) for highest slope range of 15% to 25% and low surface roughness hydrologic group.	Proposed a protocol for buffer widths for water quality and wildlife habitat functions to accommodate Intermountain West landscape attributes (northern Utah and Nevada, eastern Oregon, southwestern Montana, and Wyoming). The unadjusted widths would be subsequently adjusted to account for the presence of other buffer variables, such as wetlands, surface water features, springs, significant sand and gravel aquifers, and very steep slopes.
Buffler [65]	Recommended RBZ width ranges required for removal of selected contaminants: 20 to > 40 m for N, 3 to > 10 m for sediment, >20 m for P (dissolved and particulate), 3 to >6 m for pathogens associated with sediments, and >9 m for pesticides particulates associated with sediment.	Conducted literature review on riparian buffer width for water quality parameters within the Intermountain West and literature in other physiographic regions of the United States.
Hairston-Strang [75]	Recommended a minimum width of 11 m (35 ft) and suggested 100 m (300 ft) and wider buffers for wildlife and biodiversity.	Provided general guidance for riparian forest buffer design and maintenance strategies.
Fox, et al. [76]	Used the minimum width of grass RBZ width from 6 to 9 m (20 to 30 ft) and forest RBZ width at 11 m (35 ft).	Provided guidance on design of a riparian buffer and selection of appropriate tree and grass species.
Fischer and Fischenich [13]	Provided RBZ width recommendations based on functions of water quality protection (5–30 m), riparian habitat (30 to 500 m), stream stabilization (10 to 20 m), flood attenuation (20 to 150 m), and detrital input (3 to 10 m).	Provided general recommendations for RBZ management, suggesting a diverse array of plant species in a range of site conditions.
Dosskey, et al. [77]	Recommended minimum RBZ width from 7.6 m to 9.1 m (25–30 ft) to filter sediment, up to 30.5 m (100 ft) to provide shade, shelter, and food for aquatic organisms, and variable widths for wildlife habitat dependent upon the desired species.	Recommended minimum acceptable RBZ width depending on site conditions, vegetation type, and landowner objectives.
Dosskey, et al. [78]	Proposed a general cropland riparian buffer design consisting of a 15 m (50 ft)-wide strip of grass, shrubs, and trees between the normal bank-full water level and cropland.	Suggested the design be adjusted to better suit specific landowner needs and site conditions.
Schultz, et al. [61]	Designed a multi-species RBZ system of a 20 m wide filter strip consisting of four or five rows of fast-growing trees planted closest to the stream, two shrub rows, and a 7 m wide strip of switchgrass next to the agricultural fields.	Designed the RBZ system along a Central Iowa stream and reported better soil stabilization, absorption of infiltrated water, and lower Nitrate-nitrogen concentrations (2 mg/L) as opposed to the levels in the adjacent agricultural fields over 12 mg/L.
Isenhart, et al. [79]	Designed a multi-species RBZ. The first 10 m wide zone contained four or five rows of rapidly growing trees, the second 4 m zone contained one or two rows of shrubs, and the third 7 m zone contained native, warm-season grasses.	Reported that the buffer strips reduced sediment and chemicals by trapping over 90% of the material.
Christian [80]	Created a 21.3 m tree-shrub-grass buffer zone with a 7.3 m wide strip of a native prairie grass, switchgrass, adjacent to fields under agronomic production in Iowa. In addition, designed a 67.0 m tree-shrub-grass buffer zone along the 45.7 m grass field to address the convex-concave slope.	Analyzed the performance of RBZs at reducing sediment delivery to streams using the chemicals, runoff and erosion from agricultural management systems (CREAMS) model and reported 42% sediment reduction.
Li, et al. [81]	Created five buffer designs (10 m grass; 15 m grass; 15 m trees; 6 m grass and 24 m trees; 30 m grass) in a watershed in Pennsylvania. Simulated the nutrients and sediment loads and explored the impacts of crop rotations using the REMM along with SWAT.	Additionally, developed dynamic optimization models to investigate the necessary payoffs for farmers and landowners to adopt RBZs, suggesting that higher adoptions would be achieved with flexible buffers.

**Table A2.** Comparison of land use and precipitation between Crabtree Creek and Greens Mill Run watersheds.

Land Use	Crabtree Creek	Greens Mill Run

Urban (%)	64.6	69.6
Forested (%)	30	21
Grass/crop (%)	4.1	9.4
Precipitation (mm)	1192.8	1299.2

**Table A3.** Best fit, maximum and minimum values, and significance of performance matrices (KGE and NSE) used to calibrate: (A) Back Creek (1983–2018) and (B) Sycamore Creek and Greens Mill Run (1988–2018). The predecessor of parameters, i.e., “A” indicates the addition, “V” indicates the replacement, and “R” indicates the multiplication of the SWAT default parameter values.

A. Back Creek KGE Parameter Calibration						
Parameter Name	Description	Best Fit Value	Min. Value	Max. Value	T-Statistic	*p*-Value
A__GWQMN.gw	Threshold depth of water in the shallow aquifer required for return flow to occur	−933.08	−1000	1000	−23.7	0
A__REVAPMN.gw	Threshold depth of water in the shallow aquifer for revap to occur	−559.04	−750	750	3.67	0
V__LAT_TTIME.HRU	Lateral flow travel time	23.77	0	200	−5.96	0
A__GW_DELAY.gw	Ground water delay	−19.98	−30	90	−29.2	0
V__CANMX.HRU (FRSE, FRSD, FRST only)	Maximum canopy storage	9.11	0	25	−16.78	0
V__SLSOIL.HRU	Slope length for lateral subsurface flow	2.37	0	150	2.66	0.01
V__ALPHA_BF.gw	Baseflow alpha factor	0.9	0	1	7.52	0
V__ALPHA_BF_D.gw	Baseflow alfa factor for deep aquifer	0.7	0	1	−1.75	0.08
V__ESCO.HRU	Soil evaporation compensation factor	0.61	0.6	1	8.74	0
R__CN2.mgt	Initial SCS runoff curve number for moisture condition II	0.1	−0.1	0.1	12.34	0
A__RCHRG_DP.gw	Deep aquifer percolation fraction	0.03	−0.05	0.05	−0.06	0.95
V__GW_REVAP. gw	Ground water revap coefficient	0.02	0.02	0.1	−21.33	0
R__SOL_AWC.sol	Available water capacity of the soil layer	0.02	−0.05	0.05	0.93	0.35
**B. Sycamore Creek NSE Parameter Calibration**						
A__REVAPMN.gw	Threshold depth of water in the shallow aquifer for revap to occur	134.42	−750	750	3.05	0
A__GWQMN.gw	Threshold depth of water in the shallow aquifer required for return flow to occur	−60.77	−1000	1000	−3.48	0
A__GW_DELAY.gw	Ground water delay	29.03	−30	90	−0.39	0.7
V__CANMX.HRU (FRSE, FRSD, FRST only)	Maximum canopy storage	12.02	0	15	−16.89	0
V__SLSOIL.HRU	Slope length for lateral subsurface flow	1.33	0	150	−9.91	0
V__LAT_TTIME.HRU	Lateral flow travel time	1.01	0	14	−11.24	0
V__ALPHA_BF_D.gw	Baseflow alfa factor for deep aquifer	1	1	1	−0.17	0.87
V__ESCO.HRU	Soil evaporation compensation factor	0.94	0.6	1	123.2	0
V__ALPHA_BF.gw	Baseflow alpha factor	0.37	0	1	0.79	0.43
R__CN2.mgt	Initial SCS runoff curve number for moisture condition II	0.1	−0.1	0.1	8.24	0
V__GW_REVAP. gw	Ground water revap coefficient	0.05	0.02	0.1	−1.47	0.14
R__SOL_AWC.sol	Available water capacity of the soil layer	0.02	−0.05	0.05	−3.63	0
A__RCHRG_DP.gw	Deep aquifer percolation fraction	0.01	−0.05	0.05	−0.39	0.7

**Table A4.** Description of the LASSO climate simulation options.

Simulation Order	Location	Dataset (D)	RCP (E)	Timeframe (P)	Season (T)	Strategy (S)	Number of Options (D.E.P.T.S)

1	NC	LOCA (1)	RCP8.5 (1)	All three (3)	All five (5)	LASSO (1)	15
2	NC	BCSD (1)	RCP8.5 (1)	2070–2099 (1)	Annual (1)	LASSO (1)	1
3	NC	LOCA (1)	RCP4.5 (1)	2070–2099 (1)	Annual (1)	LASSO (1)	1
4	NC	LOCA (1)	RCP8.5 (1)	2070–2099 (1)	Annual (1)	Four Corners (1)	1
5	NC	LOCA (1)	RCP8.5 (1)	2070–2099 (1)	Annual (1)	Middle Corners (1)	1
6	NC	LOCA (1)	RCP8.5 (1)	2070–2099 (1)	Annual (1)	Double Median (1)	1
7	VA	LOCA (1)	RCP8.5 (1)	2070–2099 (1)	Annual (1)	LASSO (1)	1

## References

[1] USEPA. 2017 National Water Quality Inventory Report to Congress; U.S. Environmental Protection Agency: Washington, DC, USA, 2017.

[2] Chesters G; Schierow L-J A primer on nonpoint pollution. J. Soil Water Conserv. 1985, 40, 9–13.

[3] Ghimire SR; Johnston JM Sustainability assessment of agricultural rainwater harvesting: Evaluation of alternative crop types and irrigation practices. PLoS ONE 2019, 14, e0216452. 31075147 10.1371/journal.pone.0216452 PMC6510416

[4] Kalnay E; Cai M Impact of urbanization and land-use change on climate. Nature 2003, 423, 528–531. 12774119 10.1038/nature01675

[5] Seto KC; Güneralp B; Hutyra LR Global forecasts of urban expansion to 2030 and direct impacts on biodiversity and carbon pools. Proc. Natl. Acad. Sci. USA 2012, 109, 16083–16088. [PubMed] 22988086 10.1073/pnas.1211658109 PMC3479537

[6] Vigiak O; Malagó A; Bouraoui F; Grizzetti B; Weissteiner CJ; Pastori M Impact of current riparian land on sediment retention in the Danube River Basin. Sustain. Water Qual. Ecol. 2016, 8, 30–49.

[7] Mayer PM; Reynolds SK Jr.; McCutchen MD; Canfield TJ Meta-analysis of nitrogen removal in riparian buffers. J. Environ. Qual. 2007, 36, 1172–1180. 17596626 10.2134/jeq2006.0462

[8] Fischer RA; Martin CO; Fischenich J Improving riparian buffer strips and corridors for water quality and wildlife. In Proceedings of the International Conference on Riparian Ecology and Management in Multi-Land use Watersheds, Oregon, Portland, 28–31 August 2000.

[9] Cole LJ; Stockan J; Helliwell R Managing riparian buffer strips to optimise ecosystem services: A review. Agric. Ecosyst. Environ. 2020, 296, 106891.

[10] Pachauri RK; Allen MR; Barros VR; Broome J; Cramer W; Christ R; Church JA; Clarke L; Dahe Q; Dasgupta P Climate Change 2014: Synthesis Report. Contribution of Working Groups I, II and III to the Fifth Assessment Report of the Intergovernmental Panel on Climate Change; IPCC: Geneva, Switzerland, 2014.

[11] Mander Ü; Hayakawa Y; Kuusemets V Purification processes, ecological functions, planning and design of riparian buffer zones in agricultural watersheds. Ecol. Eng. 2005, 24, 421–432.

[12] USEPA. Riparian Buffer width, Vegetative Cover, and Nitrogen Removal Effectiveness: A Review of Current Science and Regulations; U.S. Environmental Protection Agency: Cincinnati, OH, USA, 2005.

[13] Fischer RA; Fischenich JC Design Recommendations for Riparian Corridors and Vegetated Buffer Strips; U.S. Army Corps of Engineers: Vicksburg, MS, USA, 2000.

[14] Valkama E; Usva K; Saarinen M; Uusi-Kämppä J A Meta-Analysis on Nitrogen Retention by Buffer Zones. J. Environ. Qual. 2019, 48, 270–279. 30951137 10.2134/jeq2018.03.0120

[15] Blankenberg A-GB; Skarbøvik E Phosphorus retention, erosion protection and farmers’ perceptions of riparian buffer zones with grass and natural vegetation: Case studies from South-Eastern Norway. Ambio 2020, 49, 1838–1849. 32930956 10.1007/s13280-020-01361-5 PMC7502646

[16] Mayer PM; Todd AH; Okay JA; Dwire KA Introduction to the Featured Collection on Riparian Ecosystems & Buffers1. JAWRA J. Am. Water Resour. Assoc. 2010, 46, 207–210.

[17] Vidon P; Allan C; Burns D; Duval TP; Gurwick N; Inamdar S; Lowrance R; Okay J; Scott D; Sebestyen S Hot Spots and Hot Moments in Riparian Zones: Potential for Improved Water Quality Management1. JAWRA J. Am. Water Resour. Assoc. 2010, 46, 278–298.

[18] Newbold JD; Herbert S; Sweeney BW; Kiry P; Alberts SJ Water Quality Functions of a 15-Year-Old Riparian Forest Buffer System1. JAWRA J. Am. Water Resour. Assoc. 2010, 46, 299–310.

[19] Dewalle DR Modeling Stream Shade: Riparian Buffer Height and Density as Important as Buffer Width1. JAWRA J. Am. Water Resour. Assoc. 2010, 46, 323–333.

[20] Swanson S; Kozlowski D; Hall R; Heggem D; Lin J Riparian proper functioning condition assessment to improve watershed management for water quality. J. Soil Water Conserv. 2017, 72, 168–182. 30245529 10.2489/jswc.72.2.168 PMC6145829

[21] Hoffmann CC; Kjaergaard C; Uusi-Kämppä J; Hansen HCB; Kronvang B Phosphorus Retention in Riparian Buffers: Review of Their Efficiency. J. Environ. Qual. 2009, 38, 1942–1955. 19704138 10.2134/jeq2008.0087

[22] Zhang X; Liu X; Zhang M; Dahlgren RA; Eitzel M A Review of Vegetated Buffers and a Meta-analysis of Their Mitigation Efficacy in Reducing Nonpoint Source Pollution. J. Environ. Qual. 2010, 39, 76–84. 20048295 10.2134/jeq2008.0496

[23] Uusi-Kämppä J; Braskerud B; Jansson H; Syversen N; Uusitalo R Buffer Zones and Constructed Wetlands as Filters for Agricultural Phosphorus. J. Environ. Qual. 2000, 29, 151–158.

[24] Spruill T Effectiveness of riparian buffers in controlling ground-water discharge of nitrate to streams in selected hydrogeologic settings of the North Carolina Coastal Plain. Water Sci. Technol. 2004, 49, 63–70. 15053100

[25] Cooper JR; Gilliam JW; Daniels RB; Robarge WP Riparian Areas as Filters for Agricultural Sediment1. Soil Sci. Soc. Am. J. 1987, 51, 416–420.

[26] Daniels RB; Gilliam JW Sediment and Chemical Load Reduction by Grass and Riparian Filters. Soil Sci. Soc. Am. J. 1996, 60, 246–251.

[27] Schmitt TJ; Dosskey MG; Hoagland KD Filter Strip Performance and Processes for Different Vegetation, Widths, and Contaminants. J. Environ. Qual. 1999, 28, 1479–1489.

[28] Dillaha TA; Reneau R; Mostaghimi S; Lee D Vegetative Filter Strips for Agricultural Nonpoint Source Pollution Control. Trans. ASAE 1989, 32, 0513–0519.

[29] Lowrance R; Altier LS; Newbold JD; Schnabel RR; Groffman PM; Denver JM; Correll DL; Gilliam JW; Robinson JL; Brinsfield RB; Water Quality Functions of Riparian Forest Buffers in Chesapeake Bay Watersheds. Environ. Manag. 1997, 21, 687–712. 10.1007/s002679900060 9236284

[30] Weller DE; Baker M; Jordan TE Effects of riparian buffers on nitrate concentrations in watershed discharges: New models and management implications. Ecol. Appl. 2011, 21, 1679–1695. 21830710 10.1890/10-0789.1

[31] Lyons J; Thimble SW; Paine LK Grass versus trees: Managing riparian areas to benefit streams of central north America. JAWRA J. Am. Water Resour. Assoc. 2000, 36, 919–930.

[32] Sweeney BW; Newbold JD Streamside Forest Buffer Width Needed to Protect Stream Water Quality, Habitat, and Organisms: A Literature Review. JAWRA J. Am. Water Resour. Assoc 2013, 50, 560–584.

[33] USEPA. Impaired Waters and TMDLs. Available online: https://www.epa.gov/tmdl/overview-identifying-and-restoring-impaired-waters-under-section-303d-cwa (accessed on 29 September 2020).

[34] USGPO. Clean Water Act (Federal Water Pollution Control Act). 2020, [Chapter 758 of the 80th Congress] [33 U.S.C. 1251 et seq.]. Available online: https://www.govinfo.gov/app/collection/comps/c/%7B%22pageSize%22%3A%2220%22%7D (accessed on 24 August 2021).

[35] Ghimire SR; Johnston JM Impacts of domestic and agricultural rainwater harvesting systems on watershed hydrology: A case study in the Albemarle-Pamlico river basins (USA). Ecohydrol. Hydrobiol. 2013, 13, 159–171.

[36] Ghimire SR; Johnston JM; Ingwersen WW; Hawkins TR Life Cycle Assessment of Domestic and Agricultural Rainwater Harvesting Systems. Environ. Sci. Technol. 2014, 48, 4069–4077. 24605844 10.1021/es500189f

[37] Johnston J; McGarvey D; Barber MC; Laniak G; Babendreier J; Parmar R; Wolfe K; Kraemer SR; Cyterski M; Knightes C; An integrated modeling framework for performing environmental assessments: Application to ecosystem services in the Albemarle-Pamlico basins (NC and VA, USA). Ecol. Model. 2011, 222, 2471–2484.

[38] APNEP. The Albemarle-Pamlico National Estuary Partnership. Available online: https://apnep.nc.gov/our-estuary/fast-facts (accessed on 6 May 2021).

[39] Survey, U.G. MRLC Consortium—National Land Cover Database 2001. Available online: https://www.usgs.gov/centers/eros/science/national-land-cover-database?qt-science_center_objects=0#qt-science_center_objects (accessed on 24 February 2021).

[40] USDA. Land Use—Cropland Data Layer (Agricultural). Available online: https://nassgeodata.gmu.edu/CropScape/ (accessed on 24 February 2021).

[41] Cunningham K; Stuhlinger C; Liechty H Riparian Buffers: Types and Establishment Methods; University of Arkansas, Division of Agriculture Cooperative Extension: Fayetteville, AR, USA, 2009; Available online: https://www.landcan.org/pdfs/rip%20buffer%20types.pdf (accessed on 24 August 2021).

[42] USEPA. HAWQS Version 1.2. Available online: https://new.hawqs.tamu.edu/#/ (accessed on 30 September 2020).

[43] USDA Agricultural Research Service (USDA-ARS). Texas AgriLife Research Soil & Water Assessment Tool (SWAT). Available online: https://swat.tamu.edu (accessed on 14 March 2020).

[44] Daly C; Neilson RP; Phillips DL A statistical-topographic model for mapping climatological precipitation over mountainous terrain. J. Appl. Meteorol. Climatol 1994, 33, 140–158.

[45] Arnold J; Kiniry J; Srinivasan R; Williams J; Haney E; Neitsch S SWAT 2012 Input/Output Documentation; Texas Water Resources Institute: College Station, TX, USA, 2013.

[46] TAMU. SWAT Executables. Available online: https://swat.tamu.edu/software/swat-executables/ (accessed on 26 October 2020).

[47] Winchell M; Srinivasan R; Di Luzio M; Arnold J ArcSWAT Interface for SWAT 2012; Texas A&M University: College Station, TX, USA, 2013.

[48] Neitsch SL; Arnold JG; Kiniry JR; Williams JR Soil and Water Assessment Tool Theoretical Documentation Version 2009; Texas Water Resources Institute: College Station, TX, USA, 2011.

[49] Waidler D; White M; Steglich E; Wang S; Williams J; Jones C; Srinivasan R Conservation Practice Modeling Guide for SWAT and APEX; Texas Water Resources Institute: College Station, TX, USA, 2011.

[50] USDA. State Soil Geographic Data. Available online: https://sdmdataaccess.nrcs.usda.gov/ (accessed on 5 March 2021).

[51] TAMU. HAWQS User Guide; Texas A&M University: College Station, TX, USA, 2019.

[52] Abbaspour CK SWAT calibration and uncertainty programs. A User Manual. Eawag Zur. Switz. 2015, 20, 1–100.

[53] Moriasi DN; Arnold JG; Van Liew MW; Bingner RL; Harmel RD; Veith TL Model Evaluation Guidelines for Systematic Quantification of Accuracy in Watershed Simulations. Trans. ASABE 2007, 50, 885–900.

[54] USEPA. About LASSO. Available online: https://www.epa.gov/gcx/about-lasso (accessed on 2 December 2020).

[55] USEPA. A Systematic Approach for Selecting Climate Projections to Inform Regional Impact Assessments; U.S. Environmental Protection Agency: Washington, DC, USA, 2020.

[56] IPCC. What is a GCM? Available online: https://www.ipcc-data.org/guidelines/pages/gcm_guide.html (accessed on 17 February 2021).

[57] WCRP. WCRP Coupled Model Intercomparison Project (CMIP). Available online: https://www.wcrp-climate.org/wgcm-cmip (accessed on 17 February 2021).

[58] Van Vuuren DP; Edmonds J; Kainuma M; Riahi K; Thomson A; Hibbard K; Hurtt GC; Kram T; Krey V; Lamarque J-F; The representative concentration pathways: An overview. Clim. Chang. 2011, 109, 5–31.

[59] Quinn JD; Hadjimichael A; Reed PM; Steinschneider S Can Exploratory Modeling of Water Scarcity Vulnerabilities and Robustness Be Scenario Neutral? Earth’s Futur. 2020, 8, 001650.

[60] NCDENR. Albemarle-Pamlico Baseline Water Quality Monitoring Data Summary; North Carolina Department of Environment Health, and Natural Resources: Mooresville, NC, USA, 1992.

[61] Schultz RC; Collettil JP; Isenhart TM; Simpkins WW; Mize CW; Thompson ML Design and placement of a multi-species riparian buffer strip system. Agrofor. Syst 1995, 29, 201–226.

[62] Jiang F; Gall HE; Veith TL; Cibin R; Drohan PJ Assessment of riparian buffers’ effectiveness in controlling nutrient and sediment loads as a function of buffer design, site characteristics and upland loadings. In Proceedings of the 2019 ASABE Annual International Meeting, Boston, MA, USA, 7–10 July 2019; p. 1.

[63] APNEP. Comprehensive Conservation and Management Plan (2012–2022); Albemarle-Pamlico National Estuary Partnership: Columbia, NC, USA, 2012.

[64] USDA. Conservation Reserve Enhancement Program. Available online: https://www.fsa.usda.gov/programs-and-services/conservation-programs/conservation-reserve-enhancement/index (accessed on 7 May 2021).

[65] Johnson CW; Buffler S Riparian Buffer Design Guidelines for Water Quality and Wildlife Habitat Functions on Agricultural Landscapes in the Intermountain West; U.S. Department of Agriculture, Forest Service, North Central Forest Experiment Station: Fort Collins, CO, USA, 2008; Volume 203, p. 53.

[66] Ghimire SR; Johnston JM A modified eco-efficiency framework and methodology for advancing the state of practice of sustainability analysis as applied to green infrastructure. Integr. Environ. Assess. Manag. 2017, 13, 821–831. 28304134 10.1002/ieam.1928 PMC6093199

[67] USEPA. Water Reuse Research. Available online: https://www.epa.gov/water-research/water-reuse-research (accessed on 8 December 2020).

[68] Jiang F; Preisendanz HE; Veith TL; Cibin R; Drohan PJ Riparian buffer effectiveness as a function of buffer design and input loads. J. Environ. Qual. 2020, 49, 1599–1611. 33043471 10.1002/jeq2.20149

[69] Piscoya VC; Singh VP; Cantalice JRB; Guerra SMS; Filho MC; Ribeiro CDS; Filho RNDA; Da Luz ELP. Riparian Buffer Strip Width Design in Semiarid Watershed Brazilian. J. Exp. Agric. Int. 2018, 23, 1–7.

[70] Liu C; Wu J; Clausen J; Lei T; Yang X Impact of Riparian Buffer Design on Water Quality in the Jinghe Catchment, China. In Proceedings of the 2018 ASABE Annual International Meeting, Detroit, MI, USA; 2018; p. 1.

[71] Santin F; da Silva R; Grzybowski J Artificial neural network ensembles and the design of performance-oriented riparian buffer strips for the filtering of nitrogen in agricultural catchments. Ecol. Eng. 2016, 94, 493–502.

[72] Tomer MD; Boomer KMB; Porter SA; Gelder BK; James DE; McLellan E Agricultural Conservation Planning Framework: 2. Classification of Riparian Buffer Design Types with Application to Assess and Map Stream Corridors. J. Environ. Qual. 2015, 44, 768–779. [PubMed] 26024257 10.2134/jeq2014.09.0387

[73] Cardinali A; Carletti P; Nardi S; Zanin G Design of riparian buffer strips affects soil quality parameters. Appl. Soil Ecol. 2014, 80, 67–76.

[74] Buffler S Riparian Buffer Design Guidelines for Water Quality and Wildlife Habitat Functions on Agricultural Landscapes in the Intermountain West: Appendix C; Gen. Tech. Rep. RMRS-GTR-203; U.S. Department of Agriculture, Forest Service, Rocky Mountain Research Station: Fort Collins, CO, USA, 2008; 59p.

[75] Hairston-Strang A Riparian Forest Buffer Design and Maintenance; Maryland Department of Natural Resources Forest Service: Carney, MD, USA, 2005.

[76] Fox A; Franti TG; Josiah SJ; Kucera M G05–1557 Planning Your Riparian Buffer: Design and Plant Selection; Institute of Agriculture and Natural Resources, University of Nebraska-Lincoln: Lincoln, NE, USA, 2005.

[77] Dosskey MG; Schultz RC; Isenhart TM How to Design a Riparian Buffer for Agricultural Land; Iowa State University: Ames, IA, USA, 1997.

[78] Dosskey MG; Schultz RC; Isenhart TM A Riparian Buffer Design for Cropland; Iowa State University: Ames, IA, USA, 1997; p. 6.

[79] Isenhart TM; Schultz RC; Colletti PJ Design, Function, and Management of Multi-Species Riparian Buffer Strip Systems; Iowa State University: Ames, IA, USA, 1995.

[80] Christian MF Application of the CREAMS Model to Simulate Performance of Riparian tree Buffer Strips: Implications for Buffer Strip Design; Iowa State University: Ames, IA, USA, 2018.

[81] Li X; Zipp KY; Jiang F; Veith TL; Gall HE; Royer M; Brooks R; Zikatanov LT; Shortle JS Integrated Assessment Modeling for Design of Riparian Buffer Systems and Incentives for Adoption. Available online: https://cpb-us-e1.wpmucdn.com/blogs.cornell.edu/dist/1/8608/files/2019/03/Li-et-al-2lpdbnu.pdf (accessed on 24 August 2021).

